# Outer Retinal Cell Replacement: Putting the Pieces Together

**DOI:** 10.1167/tvst.10.10.15

**Published:** 2021-11-01

**Authors:** Allison L. Ludwig, David M. Gamm

**Affiliations:** 1 Waisman Center, University of Wisconsin–Madison, Madison, WI, USA; 2 McPherson Eye Research Institute, University of Wisconsin–Madison, Madison, WI, USA; 3 School of Veterinary Medicine, University of Wisconsin–Madison, Madison, WI, USA; 4 Department of Ophthalmology and Visual Sciences, University of Wisconsin–Madison, Madison, WI, USA

**Keywords:** photoreceptor, retinal organoid, transplantation, retinal degeneration, human pluripotent stem cell

## Abstract

Retinal degenerative diseases (RDDs) affecting photoreceptors (PRs) are one of the most prevalent sources of incurable blindness worldwide. Due to a lack of endogenous repair mechanisms, functional cell replacement of PRs and/or retinal pigmented epithelium (RPE) cells are among the most anticipated approaches for restoring vision in advanced RDD. Human pluripotent stem cell (hPSC) technologies have accelerated development of outer retinal cell therapies as they provide a theoretically unlimited source of donor cells. Human PSC-RPE replacement therapies have progressed rapidly, with several completed and ongoing clinical trials. Although potentially more promising, hPSC-PR replacement therapies are still in their infancy. A first-in-human trial of hPSC-derived neuroretinal transplantation has recently begun, but a number of questions regarding survival, reproducibility, functional integration, and mechanism of action remain. The discovery of biomaterial transfer between donor and PR cells has highlighted the need for rigorous safety and efficacy studies of PR replacement. In this review, we briefly discuss the history of neuroretinal and PR cell transplantation to identify remaining challenges and outline a stepwise approach to address specific pieces of the outer retinal cell replacement puzzle.

## Introduction

**Figure 1.**
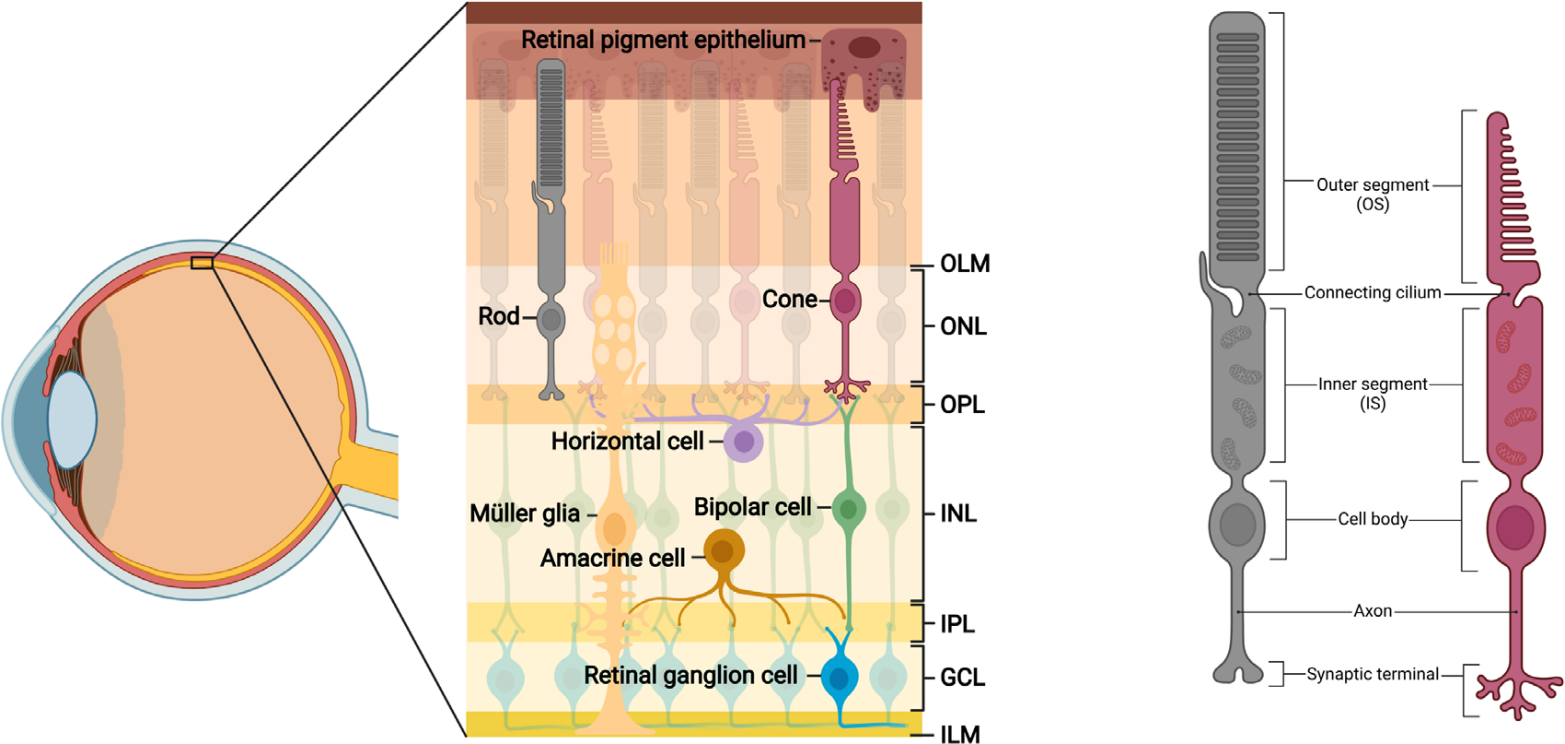
**Organization and circuitry of the retina.** (**A**) The retina contains three layers of cell bodies: the outer nuclear layer (ONL), in which rod and cone cell bodies reside; the inner nuclear layer (INL), containing horizontal cell (HC), bipolar cell (BC), amacrine cell (AC) and Müller glial (MG) cell bodies; and the ganglion cell layer (GCL) where retinal ganglion cell (RGC) somata and displaced ACs are found. PRs are supported by close apposition to the retinal pigment epithelium (RPE). The neural retina is bound apically by the outer limiting membrane (OLM) and basally by the inner limiting membrane (ILM), both formed by end-feet of the MG. PRs connect with BCs and HCs via synapses in the outer plexiform layer (OPL). The inner plexiform layer (IPL) contains signal-carrying synapses between BCs, ACs, and RGCs. (**B**) Rod and cone PRs display several distinct morphologic features. The outer segment (OS) contains stacked discs of photosensitive opsins for light detection. The connecting cilium facilitates trafficking between outer and inner segments (IS), the latter of which are rich in mitochondria. Extending from the cell body are axons with synaptic terminals, which interact with inner retinal neurons at triad ribbon synapses.

The retina is a complex tissue whose anatomy and circuitry (Fig. 1A) is predicated on the function of rod and cone photoreceptors (PRs), highly specialized neurons (Fig. 1B) that have evolved over millions of years to optimally harness light for navigating diverse environments.^1^^,^^2^ In healthy retinas, PRs are the initiators of visual activity; they are defined by their ability to capture light entering the eye and generate an electrical signal through a cascade of biochemical activity known as phototransduction.^2^ Sparking this signal is not solely enough to confer vision—PRs must also successfully relay light sensory information via synapses with retinal interneurons to begin a stepwise process of conveying visual stimuli to the brain along retinal ganglion cell (RGC) axons. The biochemical processes within PRs require extensive metabolic activity, largely mediated by their interactions with the retinal pigment epithelium (RPE).^3^ Together with Müller glia (MG), the RPE plays a crucial role in supporting PRs to maintain outer retinal structure, function, and homeostasis.^3^^–^^5^

**Figure 2.**
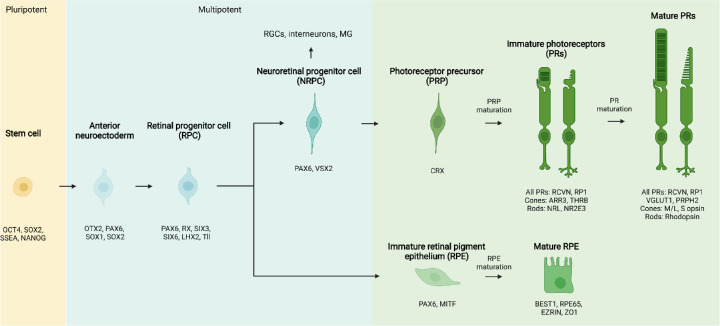
**Phases of RPE and PR differentiation.** Pluripotent stem cells pass through an anterior neuroectodermal stage to become multipotent retinal progenitor cells (RPCs), which are capable of producing all types of neuroretinal cells in addition to RPE. Neuroretinal progenitor cells (NRPCs) become further fate restricted over time and have the capacity to generate all neural retina cell types, including photoreceptor precursors (PRP). Over time, PRP and RPE mature to express several characteristic morphologic features. Examples of key transcription factors and defining cell markers for each stage are listed below each stage. Human PSC technologies follow these developmental pathways to reproducibly generate a variety of donor cells for replacement therapies.

Like all retinal cells, both PRs and RPE arise from a common retinal progenitor cell (RPC) (Fig. 2); intrinsic^6^ and extrinsic factors work in concert to guide cells through distinct developmental stages^7^^,^^8^ to reach functional maturity. In outer retinal degenerative diseases (RDDs) the interdependent nature of PRs and RPE becomes a weakness; primary dysfunction in either population often causes secondary damage in the other.^9^^–^^12^ Regardless of the inciting cause, PR damage instigates a predictable cascade of degenerative changes within the retina,^12^ progressing from widespread PR malfunction to cell death, retinal remodeling, and—in the absence of successful intervention—inner retinal neurodegeneration.^13^ Like most neurons, human PRs are nonregenerative, and these destructive processes ultimately lead to irreversible vision loss. Retinitis pigmentosa (RP) and age-related macular degeneration (AMD) are the most common inherited and acquired outer RDDs, respectively, and collectively affect millions of individuals worldwide. Blinding outer RDDs affect an increasing proportion of the global population,^14^^–^^16^ and beyond being a source of visual morbidity, can cause severe emotional distress in some individuals.^17^^,^^18^ The estimated global economic impact of potential productivity lost due to moderate and severe visual impairment is a staggering US $411 billion annually.^19^ In response, the National Eye Institute launched the Audacious Goals Initiative (AGI) in 2015 to accelerate development and deployment of ocular stem cell-based therapies for incurable RDDs.^20^^,^^21^ Specifically, the AGI aims to restore “usable vision in humans through the regeneration of neurons and neural connections” through endogenous or exogenous replacement.^20^

Gene therapies developed for individuals across the RDD spectrum have made remarkable progress in recent years^22^; however, with more than 200 distinct causative genes,^16^ curative interventions remain out of reach for most patients. Cell therapies—the delivery of live cells to treat or cure disease—have emerged as a promising alternative (or adjunct^23^) to gene therapy, offering a broad-spectrum and gene-independent strategy for restoring vision. There are currently no US Food and Drug Administration (FDA)-approved cell therapy applications for retinal disease, although several approaches have reached early phases of translational research (Box 1). Cell therapies come in many varieties, but are fundamentally characterized in the context of their source, their capacity to become other types of cells,^24^ and their purity (Table 1). Of the many donor cell sources proposed for use in RDDs, several autologous and allogeneic cell therapies have entered clinical trials.^25^^,^^26^ Human pluripotent stem cells (hPSCs) have proven to be an indispensable source of cells for such therapies as they can, theoretically, self-replicate indefinitely and form virtually any type of cell. Human PSC-based therapies advancing most rapidly toward clinical translation are aimed at supporting surviving host PRs, either through neuroprotective approaches (e.g. delivery of trophic factor-secreting stem/progenitor cells) or replacement of defective RPE.^25^^,^^26^ Exogenous replacement of PRs has progressed comparatively slowly, as it relies upon survival and integration of a sufficient number of these complex, nonreplicative, and highly specialized sensory neurons with establishment of functional synaptic connections to host interneurons.

**Box 1.** The Translational Research Continuum


Translational research aims to maximize basic science discoveries for direct application in advancing human health (also referred to as “bench-to-bedside” research). The process of bringing a new discovery to clinical practice often takes decades, and retinal cell therapies are still in the early stages of this process. Translational research is typically classified in four phases—T1 through T4 (see Zarbin, 2020^234^ for further details):
• **T1 –** scientific discovery and development from preclinical studies to phase I and II clinical trials
• **T2** – determination of efficacy in humans through phase III and IV clinical trials
• **T3** – dissemination and implementation of therapies beyond clinical trials
• **T4** – public health and policy-level assessment of established therapies
Each phase also represents a continuum of research activities. Retinal cell therapies—including RPE and PR replacement—are both currently in phase T1. RPE replacement is nearing phase T2 with several clinical trials underway, while PR replacement is largely still in preclinical development.

**Table 1.** Defining Characteristics of Donor Cell Populations



**Source**	• Autologous: patient-derived• Allogeneic: donor-derived (potentially HLA-matched and/or genetically engineered)
**Potential**	• Pluripotent: capable of forming cells from all three germ layers (e.g. human ES or iPS cell)• Multipotent: capable of forming a limited range of cell types from a common lineage (e.g. retinal progenitor cell)• Unipotent: capable of forming one cell type or class (e.g. photoreceptor precursor)
**Purity**	• Heterogenous: the cell product consists of the target cell type intermixed with multiple off-target cell types• Enriched: the cell product is predominantly comprised of the target cell type• Purified: the cell product is exclusively comprised of the target cell type

Exogenous PR replacement currently appears best poised to reach the National Eye Institute's (NEI's) audacious goal first, although several key hurdles remain as the field advances into clinical trials. With the initiation of a first-in-human safety study of hiPSC-derived neuroretinal sheets in advanced RP (RIKEN, JRCT ID jRCTa050200027),^27^ a critical appraisal of where the field currently stands with respect to PR replacement is warranted. This review summarizes relevant historic literature and highlights recent developments in exogenous hPSC-derived PR replacement therapies, identifying remaining challenges and emerging strategies. Therapies aimed at rescue of PRs have recently been reviewed elsewhere and, for the purposes of this review, are largely discussed in the context of principles relevant to PR replacement. Readers interested in PR rescue are directed to several excellent reviews of RPE replacement^27^^,^^28^ and other approaches including stem/progenitor trophic cell therapies.^29^ In addition, recent reviews of biomaterial transfer,^30^ outer retinal scaffolds,^31^^,^^32^ immune responses in subretinal cell therapies,^33^ PR replacement in rodents,^34^ and clinical trials of cell transplantation in RDDs^25^^,^^26^ may aid the reader in gaining a comprehensive understanding of the field.

## The Rise of Photoreceptor Replacement Therapy

### 1950s to 1990s: Early Studies in Primary Cell Transplantation

**Figure 3.**
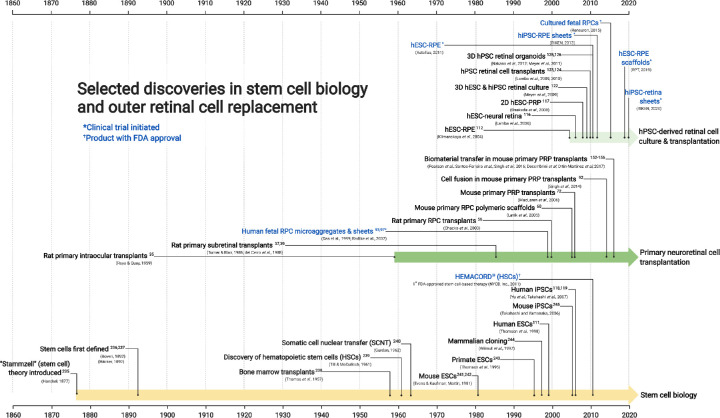
**An abbreviated history of stem cell biology and its applications to retinal cell replacement.** Selected discoveries in stem cell biology and retinal cell transplantation that have contributed to the advancement of outer retinal cell replacement therapies.

The number of published studies aimed at retinal cell replacement has increased in recent years, but the field itself is far from new. Retinal regeneration stands on the shoulders of giants in many regards, building upon more than a century of research in stem cell biology and retinal development (Fig. 3). While intraocular delivery of retinal tissue began in the late 1950s,^35^^,^^36^ proof-of-principle for neuroretinal cell replacement (i.e. retina-into-retina transplantation) was established by a series of landmark studies by del Cerro, Turner, and Blair in the late 1980s.^37^^–^^41^ Turner and Blair were the first to transplant primary retinal tissue from neonatal rats into the subretinal space (SRS) of adult rats with outer retinal lesions,^37^ reporting survival and differentiation of grafts at 4 weeks post-transplant. These experiments documented some of the earliest evidence of “integration” between donor cells and host retinal tissue, a phenomenon that would eventually grow to become a source of significant debate in retinal regenerative therapies (see Supplementary Note S1).

Retinal developmental biology saw unprecedented progress throughout the 1980s and 1990s; the introduction of cell birth dating and advanced molecular biology techniques uncovered mechanisms of cell specification, organization, and neuronal maturation within the retina.^6^^–^^8^^,^^42^ As the developmental trajectory of PRs was defined (see Fig. 2), investigators continued to experiment with primary cell isolation to determine the optimal donor stage for cell replacement.^43^^–^^45^ Those studies led to the observation that donor cell suitability for transplantation decreased with age. That is, in both allografts (i.e. same-species transplants) and xenografts (i.e. cross-species transplants), embryonic and early postnatal retinal grafts incorporated into lesioned retinas far more readily than their older counterparts.^45^^,^^46^ Given these findings, dissociated suspensions of multipotent neural^47^^–^^52^ or retinal progenitor cells^53^^–^^56^ were initially preferred by many. However, limited survival due to reflux and cell death (often less than 0.01% of the starting dose^53^^,^^57^) in addition to unpredictable differentiation led to low rates of PR engraftment, making translation to human therapies difficult.^51^

### 1990s to Early 2000s: Improving on Dissociated RPC Delivery

Two main approaches emerged to address the issue of poor engraftment. Studies building on the findings of del Cerro, Turner, and Blair suggested that human fetal RPC (fRPC) microaggregates (i.e. clusters of cells) and retinal sheets offered improved survival relative to dissociated cell transplants,^58^ likely due to enhanced structural support and maintenance of cell-cell contacts (reviewed by Seiler and Aramant, 2012). Anoikis, the anchorage-dependent death of cells following loss of extracellular matrix (ECM) contacts, was thought to play a role in the poor survival of subretinally transplanted dissociated cells.^59^ Tissue-engineered scaffolds were introduced as a customizable approach for mimicking the native structure of retinal tissue to improve survival in RPC transplants.^57^^,^^60^^–^^66^ A variety of naturally occurring gelatinous matrices, hydrogels, and decellularized tissues were initially used; however, graft organization was limited and concerns regarding batch-to-batch variability restricted future clinical use.^31^^,^^67^^–^^69^ Among others, the Young laboratory developed criteria for an ideal neuroretinal scaffold: biodegradable and/or biocompatible, optically clear, porous, flexible yet strong, and thin enough for relatively easy subretinal delivery (<50 µm).^57^^,^^60^^–^^66^ Many synthetic biomaterials met these criteria, and a variety of polymers including poly(e-caprolactone) (PCL), poly(L-lactic acid) (PLLA), poly(lactic-*co*-glycolic acid) (PLGA), poly(methyl methacrylate) (PMMA), polydimethylsiloxane (PDMS), and poly(glycerol sebacate) (PGS) were found to be well-tolerated in the SRS and supportive of improved RPC delivery in pigs and rodents.^60^^,^^62^^–^^65^^,^^70^^,^^71^ However, RPCs were not limited to producing PRs (see Fig. 2), and despite enhanced survival, the efficiency of PR engraftment following RPC scaffold delivery remained relatively low.^57^^,^^64^

The second approach—enrichment of committed PR precursors (PRPs)—was introduced by MacLaren et al. in 2006 with transplantation of green fluorescent protein (GFP)-labeled rod precursors (*Nrl-gfp^+/+^* cells) in mice.^72^ Characterized by a defined cell fate, PRPs offered substantial improvement in apparent PR engraftment in the retinas of wild type and rhodopsin-null mice. MacLaren and colleagues observed recovery of light sensitivity in rhodopsin-null mice, providing some of the earliest evidence of functional rescue following subretinal cell transplantation and sparking broad interest within scientific and lay communities alike. Although the study (and at least one subsequent report^73^) referenced fusion between donor and host cells as a potential alternative explanation for their results,^72^ it was not believed to occur to a significant degree in the retina at the time. Indeed, as a control, *Nrl-gfp^+/+^* cells were transplanted into transgenic cyan fluorescent protein (CFP) reporter mice, and on the basis of qualitative data showing a lack of multinucleate or double-labeled GFP+/CFP+ cells, MacLaren et al. argued that cell fusion—at least in the classical sense—was unlikely.

Studies within the Ali, Wallace, and Ader laboratories (among others) replicated the findings of MacLaren et al. in the years that followed*,* primarily in rodent models with an intact or partially degenerated outer nuclear layer (ONL). Manipulation of the degenerative retinal environment by disrupting potential barriers to integration—including the outer limiting membrane (OLM),^74^^,^^75^ glial scarring,^76^^,^^77^ and chondroitin sulfate proteoglycan deposition^78^^–^^80^—was proposed to further enhance PRP incorporation. Fluorescence-activated cell sorting (FACS)^72^^,^^81^^–^^83^ and magnetic-activated cell sorting (MACS)^84^^–^^86^ were optimized to obtain relatively uniform batches of transplantable cells. The developmental window paradigm, which proposed that effective PRP cell replacement is best achieved through delivery of postmitotic cells at the precise peak of PRP genesis (E15 to P4 in mice), was introduced during this time.^87^ With mounting evidence of their success in cell replacement—including improvements in light-mediated activity^76^^,^^82^^,^^88^ even in advanced degeneration^89^—PRP became the preferred developmental stage for primary cell transplantation among most investigators.^73^^,^^81^^–^^86^^,^^88^^–^^91^ As in MacLaren et al.,^72^ integration was assumed to be the predominant mechanism in these studies; however, the MacLaren laboratory first raised concerns regarding PRs double-labeled with donor and host fluorescent reporters in 2014,^92^ suggesting that fusion between donor and host cells was indeed possible.

### Limitations of Primary Cells

Both strategies—scaffolds and PRP enrichment—offered apparent improvements relative to dissociated RPC delivery, but primary cells presented major challenges to translation beyond animal studies. Phase I and II clinical trials of human fetal-derived retinal cells,^93^ microaggregates,^94^ and retinal sheets with RPE^95^^–^^97^ in advanced RP and AMD began in the late 1990s, but ultimately reported mixed effects on visual outcomes in humans. A phase II study led by Radtke and colleagues reported transient visual acuity improvement in 7 of 10 subjects with long-term stabilization in a single subject,^95^ but the study did not distinguish trophic effects from functional integration, and interpretations of the underlying mechanism varied.^26^^,^^58^^,^^98^^,^^99^ These early studies (conducted without immunosuppression) provided evidence of safety for future cell therapies, demonstrating a clear path to clinical trials through careful preclinical study planning, but the primary cell approach to replacing PRs faced a difficult road to widespread application.

Procurement of human fetal retinal tissue for transplantation proved controversial from its introduction in the early 1980s,^100^ and debate around its use in biomedical research continues.^101^ The developmental window paradigm for PRP (E15-P4 in mice) coincided with the second trimester of pregnancy in humans, presenting an ethical minefield for translation to clinical use. Attempts to expand^102^^,^^103^ and immortalize^104^ human fetal retinal cells were largely unable to circumvent the issue as RPCs were by definition not restricted to the PR lineage (see Fig. 2) and generated few PRs. In most cases, human fRPCs lost neurogenic potential over time in culture^105^^,^^106^ and demonstrated poor long-term survival following transplantation.^104^ Improvement under low-oxygen culture conditions was reported in some cases,^53^^,^^107^^–^^110^ eventually resulting in the recent initiation of a phase I/IIa clinical trial of subretinal fRPC delivery in late-stage RP (ReNeuron, clinicaltrials.gov identifier NCT02464436). Although this trial is expected to yield valuable safety and efficacy data,^26^ results have not yet been published, and difficulty in distinguishing trophic support from functional PR replacement remains.^26^ Ethical constraints and ambiguous mechanisms aside, primary cells and their derivatives continued to present a yield dilemma: with millions of potential patients,^14^^,^^16^ reproducible manufacturing was expected to be a bottleneck for larger phase III clinical trials and beyond.^26^^,^^58^

### Early 2000s to Late 2010s: Expanding Potential With Human Pluripotent Stem Cells

The isolation of human embryonic stem cells (hESCs) in 1998^111^ ushered in a new era for retinal cell replacement. The first completely in vitro differentiation of RPE was achieved in relatively short order,^112^ but PR differentiation proved more challenging. Building on existing mouse ESC protocols,^113^^,^^114^ studies by the Reubinoff and Reh laboratories showed hESCs could be guided toward a PR fate, but only when transplanted into the SRS^115^ or co-cultured with retinal tissue.^116^ Osakada et al. were the first to achieve in vitro generation of hPSC-derived PRP in the absence of mature retinal tissue^117^ in 2008. The earliest neuroretinal differentiation protocols yielded few PRP, however (just 12–20% of all cells^116^^,^^117^), and only a fraction of these expressed mature PR markers (<0.01–10% of all cells^116^^,^^117^). Induced pluripotent stem cells (iPSCs) were introduced shortly thereafter,^118^^,^^119^ and expanding on earlier approaches,^106^^,^^113^^,^^117^^,^^120^^,^^121^ our laboratory and the Takahashi laboratory soon reported successful differentiation of RPE, RPCs, and putative PRPs from both ESCs and iPSCs.^121^^,^^122^ Lamba and colleagues demonstrated that transplantation of retinal cells derived from both classes of hPSCs was feasible,^123^^,^^124^ reporting results strikingly similar to that of MacLaren et al., although donor cell survival and light responses were comparatively low.^123^^,^^124^

Protocols introduced by our laboratory and the Sasai laboratory in the early 2010s^125^^,^^126^ enabled hPSC-derived 3D retinal organoid production, overcoming the yield barriers of fetal-derived primary tissues and earlier differentiation protocols. Organoid cultures produced PRP far more efficiently—40% to 80% of all cells^122^^,^^125^—and proved to be a breakthrough technology for the field. For the first time, bulk production of PRs from a single donor source was achievable. Methods to further bias organoids toward robust PR production were refined in the years that followed,^127^^–^^129^ demonstrating a surprising degree of structural and functional authenticity^127^^,^^129^^–^^135^ (see Bell et al., 2020^133^ for further discussion). With growing evidence that PSC-derived retinal cells could serve as a reliable and reproducible source of donor cells, the field shifted toward rodent^136^^–^^143^ and human^90^^,^^144^^,^^145^ PSC-derived cells and tissues for PR replacement. The preference to use PRP-rich cells and retinal sheets^146^^–^^149^ over RPC donor material largely persisted, given the greater degree of proliferation, disorganization, and uncontrolled migration observed in transplants using the latter.^150^^,^^151^

### Late 2010s: The Paradigm-Shifting Discovery of Material Transfer

The field effectively experienced a reset with the revelation of widespread fluorescent material transfer between conspecific donor and host PRs, independently reported by several groups between 2016 and 2017.^152^^–^^156^ In a subsequent transplant study by Waldron et al., nearly all GFP+ cells (99%) found in wildtype host retinas and most GFP+ cells (>75%) in degenerative *Nrl^−^^/^^−^ and Prph2^rd2/rd2^* retinas were estimated to result from material transfer,^157^ calling the results of several previous rodent studies into question.^72^^,^^76^^,^^82^^,^^88^^,^^123^^,^^145^^,^^158^ Although the exact mechanism and longevity of this phenomenon remains to be determined, at present, several points are clear. First, material transfer is more likely to occur in degenerating retinas with remaining host PRs^152^^–^^156^ than in models of end-stage retinal degeneration.^89^^,^^141^^,^^146^^,^^147^^,^^149^^,^^159^^–^^163^ Second, a variety of PR-specific proteins (cone arrestin, opsins, and peripherin-2 [PRPH2]; see Fig. 2) as well as cytoplasmic reporters can be passed via material transfer in mice,^34^ leading to a re-evaluation of how the field identifies and defines integration (see Supplementary Note S1 for further discussion). Third, PSC-derived PRP do not appear to be exempt from this phenomenon^34^^,^^137^^,^^140^^,^^157^; however, at least one study has suggested that the capacity for material transfer is lower in human-into-rodent xenografts than in allogeneic transplantation.^144^ Finally, many of the central tenets of successful cell replacement—including the developmental window paradigm, estimated donor cell survival rates, evidence of a dose response, integration, and synaptogenesis—require re-examination in the context of material transfer.

### Lessons Learned From Historic Studies of PR Replacement

Collectively, early studies in retinal cell replacement identified several guiding principles for carrying PR cell therapies closer to the clinic. Multiple strategies, including the use of enriched donor cell populations and biomaterial-based scaffolds, have been shown to enhance cell survival in the face of low PR engraftment.^34^^,^^70^^,^^72^ The PRP stage of differentiation remains preferred for replacing PRs, although the window of transplant competence is likely not so narrow as previously estimated.^34^ Three-dimensional retinal organoids are the most often used source of authentic donor cells and tissue sheets, and have in some cases been associated with modest improvements in host retinal light sensitivity following transplantation.^26^^,^^34^ Finally, rodent PSC allografts and human-into-rodent PSC xenografts have established proof-of-concept for PR survival and anatomic engraftment following transplantation. Just as important, these studies have also identified remaining hurdles for the field to overcome. Surviving donor cells often remain disorganized within the subretinal space, and the mechanisms by which they affect host vision remain unclear. With many prior studies now known to result from material transfer rather than functional integration, there is substantial interest in the development and use of quantitative methods for assessing integration, organization, and synaptogenesis.

## Current Status and Remaining Questions for Retinal Cell Therapies

**Figure 4.**
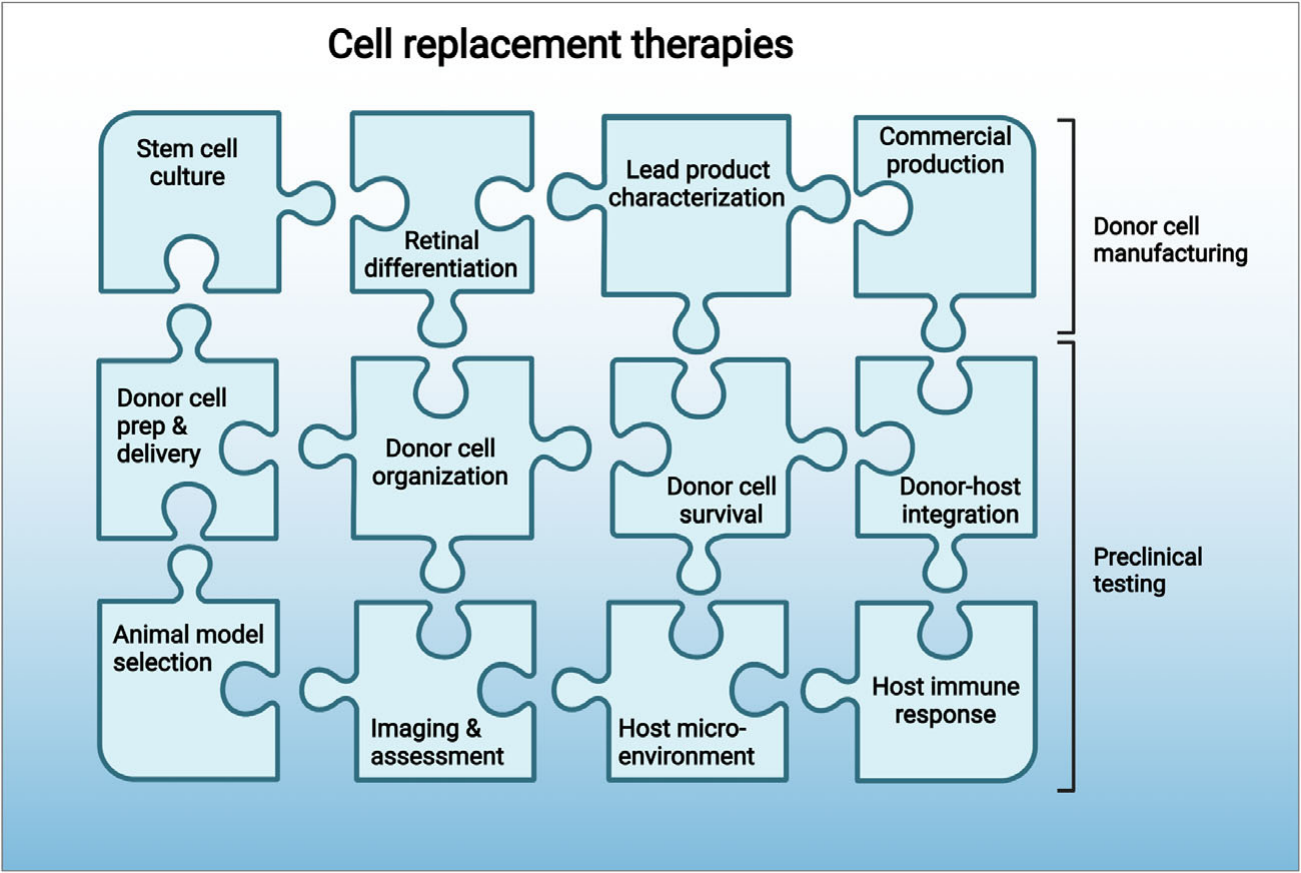
**The complex puzzle of therapeutic development for outer retinal cell therapies.** As cell therapies transition from phase T1 to phase T2 studies and beyond, several interconnected factors related to donor cell manufacturing and preclinical testing must be addressed.

As outlined above, cell replacement therapies in the retina have been studied for decades (see Fig. 3), and hPSC-RPE and hPSC-PRP cell products are now in the T1 translational research phase (which spans preclinical studies through phase II clinical trials). Thus far, the majority of clinical trials have used hESC- or hiPSC-RPE, inserted into the SRS either as dissociated cell suspensions or as monolayer sheets with or without scaffolds (see Uyama et al., 2021 for further discussion^98^). Early reports suggest that these therapies are feasible, safe, and well-tolerated in individuals with advanced retinal degenerative disease.^26^^,^^98^ However, functional outer retinal cell replacement—and more particularly PR replacement—remains a complex puzzle of cell manufacturing and preclinical testing challenges, some of which may not be fully surmountable ahead of human trials (Fig. 4). Even so, efforts to address each piece of this puzzle in a deliberate, stepwise manner would help build confidence in the potential for success. This section discusses these pieces in detail, comparing and contrasting major strategies and identifying areas where additional research is necessary to advance outer retinal cell therapeutics.

### Clinical-Grade Production

Clinical hPSC-derived cell therapies must be sterile and free from infectious agents, impurities, residual pluripotent cells, unidentified cell types, and genomic instabilities.^28^ Such criteria must be met under strict Good Manufacturing Practice (GMP)-compliant conditions^28^^,^^164^^–^^166^ and also be scalable far beyond the capacity of an average laboratory setting to be feasible for clinical trials and commercialization. Although detailed discussions of stem cell source (ES or iPS) and culture technique are beyond the scope of this review, proper induction and/or maintenance of PSCs is fundamental to any successful retinal differentiation program. Advantages and disadvantages of autologous and allogeneic cell replacement should be weighed early in product development to avoid the need for correction mid-program. Preclinical safety studies for autologous products emphasize the manufacturing process for producing each cell line in addition to the final clinical product, whereas safety studies for allogeneic products focus on the latter. Thus, programs are effectively “locked in” very early to an autologous or allogeneic approach.^28^ Autologous therapies are subject to fewer infectious disease testing requirements and are theoretically less likely to result in immune rejection,^28^ although cost (an estimated $800,000/cell line for clinical-grade iPSC production^167^) and reproducibility across patient-specific iPSC lines are often limiting factors. “Off-the-shelf” human leukocyte antigen (HLA)-matched^168^ or HLA knockout^169^ allogeneic products offer a scalable, more cost-effective production pipeline; however, immune rejection and long-term safety become a greater concern, and many dozens of HLA “super donor” cell lines would still be needed depending on the genetic diversity of the target population.^167^

Lessons learned from existing Investigational New Drug (IND)-enabling studies, particularly those from investigators with experience in cell therapies and Biologics License Applications with the FDA,^26^ should be considered early in product development to mitigate additional “known unknown” risks for scaling regenerative therapies^170^ (for further discussion of quality control for clinical-grade hPSC retinal cell production, see Wright et al., 2014^171^ and Sharma et al., 2020^28^). Current methods for generating hPSC retinal organoids are both time and labor-intensive, limiting their utility in clinical production pipelines. The use of bioreactors,^172^ microfluidics,^166^^,^^173^ and automated culture systems^27^ are all promising approaches currently under investigation for scaling clinical-grade organoid-based technologies.

### Donor Cell Enrichment

Robust methods for purifying or enriching PRP from stem cell-derived retinal organoids represent a critical bottleneck in the regenerative medicine pipeline for PR degenerative diseases.^34^ MACS and FACS-based enrichment strategies originally developed in mouse models^174^ have not yet translated into consistent success for enrichment of hPSC-PRP for transplantation,^161^ possibly due to species- or maturation stage-specific differences in PR cell surface markers. Although some groups have reported successful development of hPSC-PRP enrichment protocols, most have not been widely adopted outside individual laboratories, possibly due to low yield (<1 million cells)^175^^–^^177^ and/or suboptimal purity (40–70%) across various differentiation protocols.^135^ A fully homogenous cell product is not necessarily a prerequisite for clinical trial initiation^26^ because the FDA allows study sponsors to set their own release criteria for product purity, but a highly enriched PRP product (>80%) would be desirable. Some groups have proceeded with unsorted cell populations or retinal sheets in the absence of reproducible sorting methods,^145^^,^^149^ but residual proliferating cells (e.g. immature RPE, RPCs, etc.) often remain. Unsorted populations thus contain cells that may continue to divide, leading to PRs being outnumbered by off-target cell types,^178^ or to the development of disorganized, rosetted grafts following tissue sheet transplants.^136^^,^^146^^–^^149^^,^^179^

Human PSC reporter lines^135^^,^^177^^,^^180^^–^^183^ and viral labeling constructs^144^ have been generated as an alternative approach to enable rapid PRP enrichment via FACS. Although precedent exists for FDA allowance of biologics expressing fluorescent proteins in clinical trials^184^ (GenSight Biologics’ optogenetic GS030 [clinicaltrials.gov identifier NCT03326336] encodes a tdTomato-linked fusion protein), nontherapeutic inclusions add safety and regulatory hurdles to an already complex approval process. Sorting via cell surface markers^162^^,^^176^ (e.g. MACS) or label-free microfluidics^175^ offers a more favorable approach to obtaining enriched PRP for cell replacement. Given the current lack of consensus on optimal PRP sorting strategies, particularly for cones,^34^ further investigations of human PRP-specific cell surface markers and enrichment approaches are warranted.

### Functional Validation of Donor Cells

The advancement of hPSC-RPE therapies has been accelerated in part by the relative ease with which RPE donor cell identity and function can be validated.^28^ A battery of biomarkers and assays, including cobblestone morphology, pigmentation, transepithelial resistance (TER), photoreceptor outer segment (POS) phagocytosis, electrophysiology, apical-basal polarization, tight junction marker expression, and microvilli formation, are all indicative of healthy, maturing hPSC-RPE.^28^^,^^31^^,^^98^^,^^185^^,^^186^ Standardized methods for PRP validation have proven less straightforward, partly due to cell heterogeneity (rods; short-, medium-, and long-wavelength sensitive cones) and complexity, and partly due to the range in donor cell maturation used across studies. Neuronal age is a simple and widely used metric for estimating maturity in hPSC culture systems,^187^ and functional maturation is associated with age in hPSC-derived retinal neurons^114^^,^^117^^,^^122^ regardless of the differentiation protocol used. However, the rate of maturation is often asynchronous across RO differentiation protocols, cell lines, and even differentiation batches.^129^ Age can serve as a rough surrogate marker of maturation, but this metric provides a somewhat false sense of assurance for consistency between lines or differentiations.^129^^,^^188^ A recent study by Capowski et al. demonstrated the utility of morphology for assessing maturation, introducing a light microscopic staging system for classifying ROs.^129^ Stage 1 ROs contain RPCs, early-born inner retinal neurons, and an outer neuroblastic layer, whereas stage 2 ROs are characterized by differentiation of an abundance of PRs and inner retinal neurons. The hallmarks of stage 3 ROs are the development of PR outer segments and increased outer neuroretinal organization along with production and maturation of Müller glia and ongoing deterioration of the innermost retinal layers.^129^

Because the characteristic light-sensitive component of PRs, outer segments, often appear months past the peak of PRP genesis in ROs (stage 2, approximately day 80–120 of differentiation), development of in vitro potency assays for validating hPSC-PRP remains a high priority. One intriguing approach to this conundrum is the use of optogenetically engineered hiPSC-PRP, which have recently been shown to generate modest responses to bright light in vitro and in vivo.^163^^,^^189^ However, such a genetic modification presumes that hPSC-derived PRs cannot innately respond to light and also introduces aforementioned regulatory hurdles. In the absence of genetic modification of hPSC-PRP, other potency assays may prove useful for authenticating batches of transplantable hPSC-PRP, including examinations of cell polarity, synaptic marker expression,^190^ PR marker expression, axon outgrowth, and membrane electrophysiology.^135^

### Cell Preparation, Delivery, and Organization

A number of recent xenograft studies have demonstrated proof-of-concept for PR survival and anatomic engraftment (see Supplementary Note S1) following transplantation of dissociated hPSC-derived cell suspensions or retinal sheets in rodents^146^^,^^149^^,^^161^^–^^163^^,^^179^^,^^189^ and non-human primates (NHPs),^147^^,^^160^ using controls for biomaterial transfer. Dissociated cell injections have the advantage of being relatively simple, cost-effective, rapid, and minimally invasive, although graft organization and cell survival is often suboptimal.^34^ Retinal sheet delivery can afford a striking degree of self-organization relative to dissociated cells,^34^^,^^98^^,^^146^^,^^147^^,^^149^^,^^179^ but the surgical technique requires specialized instrumentation and is more complex and invasive than simple subretinal injections. Furthermore, rosetted PRs, formation of ectopic inner retinal laminae, and lack of apposition to host RPE remain limitations to this approach.^26^

PRs and RPE are both highly specialized cells for which apical-basal polarity plays a crucial role in function; there is thus substantial interest in cell delivery strategies supportive of donor cell organization. Polymeric retinal patches or scaffolds are among the most promising solutions for improving cell retention and 3D distribution^191^ and maintaining cell orientation.^192^^–^^194^ Added benefits also include a defined dose, targeted delivery to a discrete region, and potential for customization of scaffold size, shape, and material. Scaffold-based hPSC-RPE delivery has thus far been well-tolerated in clinical trials, and despite more complex surgical procedures, there is evidence to suggest that scaffold delivery may be achievable in an outpatient setting.^195^ Neuroretinal scaffold approaches are still in their infancy but appear to be similarly advantageous for PR replacement. Current hPSC approaches include two-photon polymerized PCL scaffolds seeded with clinical-grade RPCs^191^^,^^196^ and micromolded PGS scaffolds seeded with hPSC-PRPs.^192^^,^^193^ Both scaffolds are sterilizable, biodegradable, and have a desirable elastic and/or compressive modulus, which play a critical role in ease of surgical handling.^193^^,^^196^^,^^197^ Extensive in vivo safety testing has been performed for the former, although the use of RPCs rather than PRPs was a limiting factor in determining capacity for PR delivery. Micromolded scaffolds are capable of pre-organizing polarized PRPs even at high cell densities, although it remains to be seen whether such organization can be retained in vivo. Optimization of scaffold delivery requires the use of clinically relevant large animal models to simulate targeted subretinal scaffold implantation in the human macula. In addition to delivery of PRP-only scaffolds, envisaged applications include co-delivery of hPSC-RPE and PRPs, as replacement of both cell types will likely be necessary for individuals suffering from late-stage AMD^198^ or inherited maculopathies, such as Stargardt and Best disease.

### Assessment of Donor Cell Survival

Dissociated cell survival in RPC^199^ or allogeneic PSC-PRP transplants in rodents^143^ is extremely low (1–4%), and because these studies predate the discovery of material transfer, may be overestimated. Given the widespread use of percentages rather than discrete cell counts in published datasets, it is often difficult to obtain a true approximation of cell survival relative to the starting dose. Standardized methods for counting cells or regions of interest, like the QUANTOS workflow developed for synapse identification,^190^ will be critical for rigorously studying such outcomes. Where possible (and with appropriate controls), unbiased stereology and automated image analysis can also provide a less subjective approach to histologic analyses. Several studies have highlighted the importance of standardized cell quantification in biological research to increase reproducibility and aid comparisons between studies or across research groups.^200^^–^^202^ Greater adoption of such methods for assessing donor cell survival in PR replacement would serve the field well.

### Functional Integration and Synaptogenesis

The presence of new synaptic connections following transplantation is often inferred by pre- and postsynaptic protein co-immunolabeling or electron microscopic evidence of synaptic ribbons near donor cells.^136^^,^^147^^–^^149^^,^^160^^,^^161^^,^^179^ However, immunocytochemical evidence of synaptic marker expression does not establish a definitive causal link to observed changes in retinal function or visual behavior. Further evidence in favor of functional synaptogenesis includes electrophysiologic, reflexive, and behavioral assessments of light responsivity, although most of these readouts measure processes several synapses downstream from presumptive donor-host contacts.^23^^,^^146^^,^^147^^,^^149^^,^^159^^,^^161^^,^^163^^,^^179^ High levels of donor cell disorganization^26^^,^^34^^,^^136^^,^^141^^,^^146^ and relatively mild degrees of light-induced response recovery observed in most hPSC-PRP transplants^23^^,^^141^^,^^146^^,^^159^^,^^161^^,^^163^^,^^203^ also suggest that synapse formation likely occurs at lower rates than previously predicted.^34^

It is currently difficult to fully distinguish bona fide synaptic connections from existing ones—however rare they may be—in the absence of direct and effective methods for studying synaptic contacts of donor cells.^34^^,^^204^ A recent study by Cowan et al. suggests that PRP are capable of forming functional synapses within retinal organoids as evidenced by calcium imaging.^134^ However, no study to date has definitively shown that hPSC-PRPs can form new functional synapses after being isolated from retinal organoids. Evidence of functional post-transplant synaptogenesis currently includes modest light responses recorded with multi-electrode array (MEA) and micro-electroretinography (mERG),^146^^,^^147^^,^^161^^,^^189^^,^^205^ and often does not conclusively distinguish light-induced donor cell responses from possible neuroprotective effects on residual host retinal circuitry. Reproducible, well-controlled approaches for assessing de novo synaptogenesis at the level of individual donor hPSC-PRPs (via calcium imaging or viral monosynaptic circuit tracing), particularly in the context of xenogeneic transplantation,^206^ will be necessary to further clarify mechanisms of functional recovery. The efficiency of synaptogenesis in xenografts is currently unknown^206^; however, by increasing PRP survival, alignment, and organization, it may be possible to increase the likelihood of synapse formation between donor and host cells. Strategies to directly measure hPSC-PRP synaptic contacts via trans-synaptic tracing or patch-clamp recordings have been highlighted as crucial,^34^ but have not yet come to fruition.

### Animal Model Selection for Safety and Efficacy Studies

Rodents have historically been the preferred model system for retinal cell replacement studies due to cost, ease of genetic manipulation, and widespread availability. Several reports have shown that transplanted hPSC-PRP can survive and be associated with varying degrees of light-evoked behavior and/or electrophysiologic activity in degenerating rodent retinas,^146^^,^^149^^,^^161^^–^^163^^,^^179^^,^^189^ but there is not yet direct evidence of causation. The well-documented neuroprotection caused by virtually any subretinally transplanted material (including control vehicles^110^^,^^207^) in the Royal College of Surgeons (RCS) rat makes it highly difficult to fully control for alternate mechanisms in this model. To address the confounding variables of neuroprotection and biomaterial transfer, many investigators have instead opted for models with near-complete PR loss. Models with severe PR loss^89^^,^^150^^,^^159^^,^^208^ are currently considered most appropriate for studying functional integration, although even these models are not free of confounding variables, because residual cones remain in severely atrophic models like the *rd1* mouse and *S334ter-3* rat.^26^^,^^206^

To date, most available data regarding cell survival and effects on vision are skewed toward rod-dominant rodent models, although there is some evidence to suggest similar responses are possible in NHPs.^147^ Given notable species-specific differences in PR development and synaptic architecture,^206^ the degree to which these observations will directly translate to human allogeneic or autologous transplants remains to be seen. The introduction of scaffolds and more complex tissue constructs, which necessarily includes more complicated surgeries, will require a shift toward larger animal models with ocular anatomy more akin to that of humans to provide meaningful assessments of such approaches. Development of translation-enabling models that faithfully recapitulate aspects of human RDDs is an explicit aim of the NEI AGI,^21^ and these models will be a valuable resource for advancing retinal cell therapies. There is substantial interest in allogeneic transplantation of same-species PSC-derived retinal cells in parallel with xenogeneic studies, as this approach can potentially provide extrapolatable insight into the degree of functional restoration that may be reasonably expected in human clinical trials. Continued observance of field standards for defining integration (see Supplementary Note S1 for further discussion) and development of protocols to generate retinal organoids from additional laboratory model species will be essential to such activities.

Although a variety of reflexive and behavioral assays are available for assessing visual function, even electrophysiologic methods ultimately may not be sensitive enough to directly assay PR transplant-driven responses.^161^^,^^205^ Several such studies have documented light-evoked electrophysiologic responses^209^ and visual behavior^210^ in degenerating retinas even when surviving donor PRs are nearly absent. Adequately powered studies controlling for alternate explanations of restored function—including material transfer to host interneurons,^206^ aberrant firing of intrinsically photosensitive RGCs (ipRGCs), and neuroprotection of remaining host retinal cells—will be challenging, but necessary, for definitively establishing a causal link between anatomic integration and vision rescue.^26^^,^^206^

There is no single animal model that is perfect for each cell replacement application. Rather, a variety of factors, including ocular anatomy, nocturnal versus diurnal activity (i.e. rod versus cone-dominance), and genetic causation should be taken into consideration when designing preclinical IND-enabling studies for cell therapies (summarized in Table 2; also see Winkler et al. 2020^211^ for a discussion of RDD animal models). A recent study by Laver and Matsubara also suggests that the lack of robust responses observed in human-to-rodent xenografts^147^^,^^161^^,^^163^ may be due in part to synapse incompatibilities between donor PRP and host retinal interneurons.^206^ The degree of divergence in synaptic proteins between humans and non-human model organisms is just one of many factors to consider when selecting preclinical models for testing functional effects of hPSC-PRP therapeutics.

**Table 2.** Animal RDD Models and Factors Affecting Suitability for Preclinical Retinal Cell Replacement^a^

Species	Ocular Anatomy: Similarity to Human	Features	Predicted TRS^b^ Compatibility with Human^c^	Options for Immune Suppression	Selected RDD Models

Mouse	+	- Small globe with large lens	89%	Genetically modified	*Rd1, Rd10*, many others
		- Rod-dominant retina		Pharmacologic	
Rat	+	- Small globe with large lens	88%	Genetically modified	*RCS, S334ter, P23H*
		- Rod-dominant retina		Pharmacologic	
Ground squirrel	++	- Small globe with small lens	44%^d^	Pharmacologic	Retinal detachment
		- Cone-dominant retina			
Rabbit	++	- Medium-sized globe with small lens	86%	Pharmacologic	RHO (P347L)^246^
		- Visual streak			Laser damage^247^
Cat	+++	- Medium-sized globe with small lens	92%	Pharmacologic	*RDH5, CEP290, AIPL1*
		- Area centralis			
Dog	+++	- Moderately large globe with small lens - Area centralis	81%	Pharmacologic	*RHO, RPE65, PDE6A, PDE6B, SAG, ABCA4*
Pig	++++	- Large globe with small lens	85%	Pharmacologic	*RHO (P23H)*
		- Visual streak		Genetic models^248^	Laser damage
				Surgically-induced^224^	
Macaque	+++++	- Large globe with small lens	98%	Pharmacologic	*PDE6C, BBS7*
		- Macula			Laser damage

a A summary of findings from: Stanzel et al., 2019^249^ (ocular anatomy, RDD models), Laver and Matsubara, 2017^206^ (xenograft compatibility), and Winkler et al., 2020^211^ (RDD models).

b Photoreceptor triad ribbon synapse.

c Based on the Pikachurin sequence similarity (percentage) between humans and the listed species.

d Laver and Matsubara broadly refer to squirrels; this may not directly reflect TRS compatibility of specific models (e.g. 13-lined ground squirrels).

### Noninvasive Imaging to Assess Therapeutic Efficacy

The retina is a highly organized, laminated structure that has evolved to maximally harness light entering the eye.^212^ Recent advances in noninvasive retinal imaging have capitalized on these features to provide increasingly detailed pictures of in vivo retinal architecture.^213^^–^^216^ Both the NEI AGI and the Monaciano Consortium have highlighted a relative lack of rigorous, reproducible ocular imaging as a potential bottleneck in advancing clinical trials.^20^^,^^21^^,^^217^ Several recent studies have demonstrated the utility of noninvasive imaging for comprehensively studying integration and therapeutic efficacy in hPSC-PRP cell therapies.^160^^,^^218^ The Singh laboratory at Johns Hopkins identified quantifiable biomarkers for tracking fluorescent mouse cells after transplantation, developing a scoring system for multimodal confocal scanning laser ophthalmoscopy (cSLO) imaging.^218^ Several properties, including fluorescence size and intensity, graft placement, lamination, and peri-retinal proliferation, were scored longitudinally, facilitating long-term tracking of individual grafts over time. Similarly, Aboualizadeh et al. recently used fluorescence adaptive optics scanning light ophthalmoscopy (FAOSLO) to follow individual PRs in vivo in a laser-damage NHP model of PR loss.^160^ These types of correlative studies augment histologic assessment of efficacy, although further research is necessary to determine how to translate these imaging techniques to clinical trials and commercial products and how to distinguish donor cells from host biomaterial transfer in vivo. As mentioned earlier, fluorescent reporters are not necessarily prohibited in clinical trials, but development of high-resolution, label-free, noninvasive methods for tracking migration and integration of donor cells is preferable.

### Modulating Retinal Microenvironment and Immune Response

The ideal cell replacement toolbox will likely include approaches for priming the degenerative host retina for enhanced integration. Although there is evidence of some efficacy following hPSC-PRP delivery even in end-stage retinal degeneration—suggesting that host inner retinal circuitry remains viable for a time—the exact window of opportunity for effective cell replacement is currently unknown.^34^^,^^219^ Treatments being studied seek to modulate a variety of naturally occurring processes that may act as barriers to donor PR integration in the degenerate outer retina, including glial scarring,^220^ interneuron plasticity,^221^ and neurite outgrowth,^222^ which may in turn help create a more donor cell-receptive environment. Basic discovery research to better understand the molecular mechanisms involved in retinal circuit assembly, disassembly, and re-assembly will also be essential to address host-centered barriers to neuronal replacement.^20^^,^^223^

While the eye is historically considered to be immune-privileged, current evidence suggests that this privilege is relative rather than absolute, and is perhaps lost in the course of disease.^33^ Preclinical xenografts require immunodeficient hosts^46^^,^^146^^,^^150^^,^^224^ or immunosuppressive regimens,^147^^,^^160^^,^^225^ but allograft studies and clinical trials to date report conflicting evidence regarding the degree of immune suppression necessary for long-term donor cell survival. Recent studies suggest that the immunogenicity of hPSC-derived retinal tissues may actually be relatively low, and hPSC-derived retinal cells might even confer a degree of local immune suppression.^226^ As methods for assessing graft survival improve, further research regarding the role of the immune system in xenografts, allografts, and autografts will be necessary to predict best practices. Reports from hPSC-RPE clinical trials, which use a variety of immune suppressive regimens, will be highly informative for designing future clinical trials aimed at outer retinal cell replacement.^26^^,^^33^

## Conclusions: A Shared Responsibility

Exogenous RPE and PR transplantation efforts—bolstered by decades of research in regenerative medicine and retina developmental biology—have overcome significant hurdles in recent years and are now being tested in clinical trials. Although hPSC-RPE therapies are further along, remaining challenges to clinical translation for hPSC-PRP include scaling clinical-grade cell production, creating organized grafts, addressing synapse formation and functional integration, and optimizing safety and efficacy outcomes in relevant model systems.

Singh et al. recently observed that, as these challenges are met and retinal cell therapies reach early phase clinical trials, peer-reviewed interim reports may have unintended ripple effects on patients and lay audiences.^26^ Eye-catching headlines rarely reflect the nuance of underlying research findings and further fuel unrestrained public desire for stem cell-based therapies. Preclinical research can often inadvertently elicit similar responses when reports of vision restoration in animal models are picked up by the media. The current landscape of milestone-oriented funding and open-source science necessitates timely publication of results, but Singh et al. stress the importance of appropriately powered, long-term follow-up to mitigate “scientifically unfounded over-optimism” within the non-scientific community. Recent case reports have underscored the grave impacts^227^^–^^229^ of clinics prematurely capitalizing on this enthusiasm and preying on patient hope^230^ with unregulated stem cell treatments.

A recent *Lancet* commission on regenerative medicine argues that the shift from “small-scale bespoke experimental interventions” to bona fide clinical application of hPSC-based therapies will require “substantial rethinking of the social contract that supports such research and clinical practice in the public arena.” The commission contends that improving four areas—science, funding models, governance, and public/patient engagement—can prevent erosion of public trust and bridge the gap between patient expectations and currently available therapies.^231^ While it is clear that tremendous scientific progress has been made toward outer retinal cell replacement, transitioning from bench to bedside will require substantial engagement from a variety of stakeholders regarding economic burden,^21^ international governance,^27^ and public/patient interaction.^26^

The challenges that lie ahead for outer retinal cell therapies can be overcome, and the recent advances highlighted in this review suggest that the future for retinal regenerative medicine is bright. However, translation to clinical application will require considerable investment of time and scientific effort from public and private entities alike. Moreover, the necessary focus on safety in early phase research means that efficacy in human subjects, who will necessarily be at the severe end of the disease spectrum, will likely be modest at first. In short, the reality we collectively face is that translating cell therapies to effective clinical practice will take time, and for families currently battling vision loss, it will rarely feel like progress comes fast enough. In the interim, scientists and clinicians will continue to play a crucial role in right-sizing public expectations and encouraging patients to make informed decisions regarding stem cell treatments. To this end, several organizations have developed educational materials geared toward a lay audience that are freely available to share with individuals considering stem cell therapies. Materials from the International Society for Stem Cell Research, including the *Patient Handbook on Stem Cell Therapies*^232^ (available in 12 languages) and disease-specific fact sheets,^233^ as well as the McPherson Eye Research Institute's similarly themed “10 Things to Know Before You Fall Victim to a Retinal Stem Cell Scam” (see Supplementary Note S2) can help patients navigate stem cell claims while researchers around the world continue to work toward solving the complex puzzle of outer retinal cell replacement.

## Supplementary Material

Supplement 1

## References

[1] Baden T, Osorio D. The Retinal Basis of Vertebrate Color Vision. *Annu Rev Vis Sci*. 2019; 5(1): 177–200. 31226010 10.1146/annurev-vision-091718-014926

[2] Lamb TD. Evolution of the genes mediating phototransduction in rod and cone photoreceptors. *Prog Retin Eye Res*. Published online November 29, 2019: 100823, doi:10.1016/j.preteyeres.2019.100823 31790748

[3] George SM, Lu F, Rao M, Leach LL, Gross JM. The retinal pigment epithelium: Development, injury responses, and regenerative potential in mammalian and non-mammalian systems. *Progress in Retinal and Eye Research*. Published online April 23, 2021: 100969, doi:10.1016/j.preteyeres.2021.100969 33901682 PMC8536801

[4] Bringmann A, Iandiev I, Pannicke T, et al. Cellular signaling and factors involved in Müller cell gliosis: neuroprotective and detrimental effects. *Prog Retin Eye Res*. 2009; 28(6): 423–451. 19660572 10.1016/j.preteyeres.2009.07.001

[5] Reichenbach A, Bringmann A. New functions of Müller cells. *Glia*. 2013; 61(5): 651–678. 23440929 10.1002/glia.22477

[6] Cepko C. Intrinsically different retinal progenitor cells produce specific types of progeny. *Nat Rev Neuroscie*. 2014; 15(9): 615–627. 10.1038/nrn3767 25096185

[7] Swaroop A, Kim D, Forrest D. Transcriptional regulation of photoreceptor development and homeostasis in the mammalian retina. *Nat Rev Neurosci*. 2010; 11(8): 563–576. 20648062 10.1038/nrn2880 PMC11346175

[8] Brzezinski JA, Reh TA. Photoreceptor cell fate specification in vertebrates. *Development*. 2015; 142(19): 3263–3273. 26443631 10.1242/dev.127043 PMC4631758

[9] Sparrow JR, Hicks D, Hamel CP. The Retinal Pigment Epithelium in Health and Disease. *Curr Mol Med*. 2010; 10(9): 802–823. 21091424 10.2174/156652410793937813 PMC4120883

[10] Jones BW, Pfeiffer RL, Ferrell WD, Watt CB, Marmor M, Marc RE. Retinal remodeling in human retinitis pigmentosa. *Exp Eye Res*. 2016; 150: 149–165. 27020758 10.1016/j.exer.2016.03.018 PMC5031517

[11] Jones BW, Marc RE, Pfeiffer RL. Retinal Degeneration, Remodeling and Plasticity. In: Kolb H, Fernandez E, Nelson R, eds. *Webvision: The Organization of the Retina and Visual System*. Salt Lake City, UT: University of Utah Health Sciences Center; 1995. Accessed August 26, 2021, http://www.ncbi.nlm.nih.gov/books/NBK482309/.

[12] Jones BW, Marc RE. Retinal remodeling during retinal degeneration. *Exp Eye Res*. 2005; 81(2): 123–137. 15916760 10.1016/j.exer.2005.03.006

[13] Pfeiffer RL, Marc RE, Jones BW. Persistent remodeling and neurodegeneration in late-stage retinal degeneration. *Prog Retin Eye Res*. 2020; 74: 100771. 31356876 10.1016/j.preteyeres.2019.07.004 PMC6982593

[14] Wong WL, Su X, Li X, et al. Global prevalence of age-related macular degeneration and disease burden projection for 2020 and 2040: a systematic review and meta-analysis. *Lancet Glob Health*. 2014; 2(2): e106–e116. 25104651 10.1016/S2214-109X(13)70145-1

[15] Flaxman SR, Bourne RRA, Resnikoff S, et al. Global causes of blindness and distance vision impairment 1990-2020: a systematic review and meta-analysis. *The Lancet Glob Health*. 2017; 5(12): e1221–e1234. 29032195 10.1016/S2214-109X(17)30393-5

[16] Verbakel SK, van Huet RAC, Boon CJF, et al. Non-syndromic retinitis pigmentosa. *Prog Retin Eye Res*. 2018; 66: 157–186. 29597005 10.1016/j.preteyeres.2018.03.005

[17] van der Aa HPA, Comijs HC, Penninx BWJH, van Rens GHMB, van Nispen RMA. Major Depressive and Anxiety Disorders in Visually Impaired Older Adults. *Invest Ophthalmol Vis Sci*. 2015; 56(2): 849–854. 25604690 10.1167/iovs.14-15848

[18] Taylor DJ, Jones L, Binns AM, Crabb DP. ‘You've got dry macular degeneration, end of story’: a qualitative study into the experience of living with non-neovascular age-related macular degeneration. *Eye*. 2020; 34(3): 461–473. 31118490 10.1038/s41433-019-0445-8 PMC7042256

[19] Marques AP, Ramke J, Cairns J, et al. Global economic productivity losses from vision impairment and blindness. *EClinicalMedicine*. 2021; 35: 100852. 33997744 10.1016/j.eclinm.2021.100852 PMC8093883

[20] Gamm DM, Wong R. Panelists and the AW. Report on the National Eye Institute Audacious Goals Initiative: Photoreceptor Regeneration and Integration Workshop. *Trans Vis Sci Tech*. 2015; 4(6): 2. 10.1167/tvst.4.6.2 PMC4654223 26629398

[21] Becker SM, Wright CB. Update on the Status and Impact of the National Eye Institute Audacious Goals Initiative for Regenerative Medicine. *J Ocul Pharmacol Therapeut*. 2021; 37(3): 144–146. 10.1089/jop.2020.0015 PMC8060710 32877259

[22] Botto C, Rucli M, Tekinsoy MD, Pulman J, Sahel J-A, Dalkara D. Early and late stage gene therapy interventions for inherited retinal degenerations. *Prog Retin Eye Res*. Published online May 29, 2021: 100975, doi:10.1016/j.preteyeres.2021.100975 34058340

[23] Barnea-Cramer AO, Singh M, Fischer D, et al. Repair of Retinal Degeneration following Ex Vivo Minicircle DNA Gene Therapy and Transplantation of Corrected Photoreceptor Progenitors. *Molec Ther*. 2020; 28(3): 830–844. 32027843 10.1016/j.ymthe.2020.01.023 PMC7054814

[24] Gene & Cell Therapy FAQs | ASGCT - American Society of Gene & Cell Therapy | ASGCT - American Society of Gene & Cell Therapy. Accessed June 24, 2021, https://asgct.org/education/more-resources/gene-and-cell-therapy-faqs.

[25] Wang Y, Tang Z, Gu P. Stem/progenitor cell-based transplantation for retinal degeneration: a review of clinical trials. *Cell Death Dis*. 2020; 11(9): 1–14. 32968042 10.1038/s41419-020-02955-3 PMC7511341

[26] Singh MS, Park SS, Albini TA, et al. Retinal stem cell transplantation: Balancing safety and potential. *Prog Retin Eye Res*. 2020; 75: 100779. 31494256 10.1016/j.preteyeres.2019.100779 PMC7056514

[27] Maeda T, Sugita S, Kurimoto Y, Takahashi M. Trends of Stem Cell Therapies in Age-Related Macular Degeneration. *J Clin Med*. 2021; 10(8): 1785. 33923985 10.3390/jcm10081785 PMC8074076

[28] Sharma R, Bose D, Maminishkis A, Bharti K. Retinal Pigment Epithelium Replacement Therapy for Age-Related Macular Degeneration: Are We There Yet? *Ann Rev Pharmacol Toxicol*. 2020; 60(1): 553–572. 31914900 10.1146/annurev-pharmtox-010919-023245 PMC8783375

[29] Canto-Soler V, Flores-Bellver M, Vergara MN. Stem Cell Sources and Their Potential for the Treatment of Retinal Degenerations. *Invest Ophthalmol Vis Sci*. 2016; 57(5): ORSFd1–ORSFd9. 27116661 10.1167/iovs.16-19127 PMC6892419

[30] Nickerson PEB, Ortin-Martinez A, Wallace VA. Material Exchange in Photoreceptor Transplantation: Updating Our Understanding of Donor/Host Communication and the Future of Cell Engraftment Science. *Front Neural Circuits*. 2018; 12: 17. 29559897 10.3389/fncir.2018.00017 PMC5845679

[31] Hunt NC, Hallam D, Chichagova V, Steel DH, Lako M. The Application of Biomaterials to Tissue Engineering Neural Retina and Retinal Pigment Epithelium. *Advanced Healthcare Materials*. 2018; 7(23): 1800226. 10.1002/adhm.201800226 30175520

[32] Jemni-Damer N, Guedan-Duran A, Fuentes-Andion M, et al. Biotechnology and Biomaterial-Based Therapeutic Strategies for Age-Related Macular Degeneration. Part II: Cell and Tissue Engineering Therapies. *Front Bioeng Biotechnol*. 2020; 8: 588014. 33363125 10.3389/fbioe.2020.588014 PMC7758210

[33] Petrash CC, Palestine AG, Canto-Soler MV. Immunologic Rejection of Transplanted Retinal Pigmented Epithelium: Mechanisms and Strategies for Prevention. *Front Immunol*. 2021; 12: 621007. 34054796 10.3389/fimmu.2021.621007 PMC8153373

[34] Gasparini SJ, Llonch S, Borsch O, Ader M. Transplantation of photoreceptors into the degenerative retina: Current state and future perspectives. *Prog Retin Eye Res*. Published online November 13, 2018, doi:10.1016/j.preteyeres.2018.11.001 30445193

[35] Royo PE, Quay WB. Retinal transplantation from fetal to maternal mammalian eye. *Growth*. 1959; 23: 313–336. 14439778

[36] del Cerro M, Gash DM, Rao GN, Notter MF, Wiegand SJ, Gupta M. Intraocular retinal transplants. *Invest Ophthalmol Vis Sci*. 1985; 26(8): 1182–1185. 3874851

[37] Turner JE, Blair JR. Newborn rat retinal cells transplanted into a retinal lesion site in adult host eyes. *Brain Res*. 1986; 391(1): 91–104. 3955383 10.1016/0165-3806(86)90011-8

[38] Blair JR, Turner JE. Optimum conditions for successful transplantation of immature rat retina to the lesioned adult retina. *Devel Brain Res*. 1987; 36(2): 257–270. 10.1016/0165-3806(87)90029-0 3690336

[39] del Cerro M, Notter MF, Grover DA, Gash DM, Jiang LQ, del Cerro C. Chapter 16 Retinal transplants into adult eyes affected by phototoxic retinopathy. In: Gash DM, Sladek JR, eds. *Progress in Brain Research*. Vol. 78. New York, NY: Elsevier; 1988: 125–130. 10.1016/s0079-6123(08)60275-7 3266799

[40] Del Cerro M, Notter M, Wiegand S, Jiang L, del Cerro C. Replacement of rod cells into adult eyes affected by late-state phototoxic retinopathy by transplantation of developing retinal cells. *J Neur Transpl*. 1989; 1: 1–10.

[41] Turner JE, Seiler M, Aramant R, Blair JR. Chapter 17 Embryonic retinal grafts transplanted into the lesioned adult rat retina. In: Gash DM, Sladek JR, eds. *Progress in Brain Research*. Vol. 78. New York, NY: Elsevier; 1988: 131–139. 10.1016/s0079-6123(08)60276-9 3247418

[42] Cepko CL, Austin CP, Yang X, Alexiades M, Ezzeddine D. Cell fate determination in the vertebrate retina. *Proc Natl Acad Sci USA*. 1996; 93(2): 589–595. 8570600 10.1073/pnas.93.2.589 PMC40096

[43] Gouras P, Du J, Gelanze M, et al. Survival and Synapse Formation of Transplanted Rat Rods. *J Neur Transplant Plasticity*. 1991; 2(2): 91–100. 10.1155/NP.1991.91 PMC2565093 1747394

[44] Gust J, Reh TA. Adult Donor Rod Photoreceptors Integrate into the Mature Mouse Retina. *Invest Ophthalmol Vis Sci*. 2011; 52(8): 5266–5272. 21436277 10.1167/iovs.10-6329 PMC3176050

[45] Aramant R, Seiler M, Turner JE. Donor age influences on the success of retinal grafts to adult rat retina. *Invest Ophthalmol Vis Sci*. 1988; 29(3): 498–503. 3343107

[46] Aramant RB, Seiler MJ. Human Embryonic Retinal Cell Transplants in Athymic Immunodeficient Rat Hosts. *Cell Transplant*. 1994; 3(6): 461–474. 7881758 10.1177/096368979400300603

[47] Francis PJ, Wang S, Zhang Y, et al. Subretinal transplantation of forebrain progenitor cells in nonhuman primates: survival and intact retinal function. *Invest Ophthalmol Vis Sci*. 2009; 50(7): 3425–3431. 19234356 10.1167/iovs.08-2908 PMC2826708

[48] Wang S, Girman S, Lu B, et al. Long-term vision rescue by human neural progenitors in a rat model of photoreceptor degeneration. *Invest Ophthalmol Vis Sci*. 2008; 49(7): 3201–3206. 18579765 10.1167/iovs.08-1831 PMC3055787

[49] Gamm DM, Wang S, Lu B, et al. Protection of visual functions by human neural progenitors in a rat model of retinal disease. *PLoS One*. 2007; 2(3): e338. 17396165 10.1371/journal.pone.0000338 PMC1828619

[50] Van Hoffelen SJ, Young MJ, Shatos MA, Sakaguchi DS. Incorporation of murine brain progenitor cells into the developing mammalian retina. *Invest Ophthalmol Vis Sci*. 2003; 44(1): 426–434. 12506105 10.1167/iovs.02-0269

[51] Young MJ, Ray J, Whiteley SJ, Klassen H, Gage FH. Neuronal differentiation and morphological integration of hippocampal progenitor cells transplanted to the retina of immature and mature dystrophic rats. *Mol Cell Neurosci*. 2000; 16(3): 197–205. 10995547 10.1006/mcne.2000.0869

[52] Takahashi M, Palmer TD, Takahashi J, Gage FH. Widespread Integration and Survival of Adult-Derived Neural Progenitor Cells in the Developing Optic Retina. *Molec Cell Neurosci*. 1998; 12(6): 340–348. 9888988 10.1006/mcne.1998.0721

[53] Klassen HJ, Ng TF, Kurimoto Y, et al. Multipotent retinal progenitors express developmental markers, differentiate into retinal neurons, and preserve light-mediated behavior. *Invest Ophthalmol Vis Sci*. 2004; 45(11): 4167–4173. 15505071 10.1167/iovs.04-0511

[54] Qiu G, Seiler MJ, Mui C, et al. Photoreceptor differentiation and integration of retinal progenitor cells transplanted into transgenic rats. *Exp Eye Res*. 2005; 80(4): 515–525. 15781279 10.1016/j.exer.2004.11.001

[55] Akagi T, Haruta M, Akita J, Nishida A, Honda Y, Takahashi M. Different characteristics of rat retinal progenitor cells from different culture periods. *Neurosci Lett*. 2003; 341(3): 213–216. 12697286 10.1016/s0304-3940(03)00177-0

[56] Chacko DM, Rogers JA, Turner JE, Ahmad I. Survival and Differentiation of Cultured Retinal Progenitors Transplanted in the Subretinal Space of the Rat. *Biochem Biophysic Res Commun*. 2000; 268(3): 842–846. 10.1006/bbrc.2000.2153 10679293

[57] Tucker BA, Redenti SM, Jiang C, et al. The use of progenitor cell/biodegradable MMP2-PLGA polymer constructs to enhance cellular integration and retinal repopulation. *Biomaterials*. 2010; 31(1): 9–19. 19775744 10.1016/j.biomaterials.2009.09.015

[58] Seiler MJ, Aramant RB. Cell replacement and visual restoration by retinal sheet transplants. *Prog Retin Eye Res*. 2012; 31(6): 661–687. 22771454 10.1016/j.preteyeres.2012.06.003 PMC3472113

[59] Lund RD, Ono SJ, Keegan DJ, Lawrence JM. Retinal transplantation: progress and problems in clinical application. *J Leukocyte Biol*. 2003; 74(2): 151–160. 12885930 10.1189/jlb.0103041

[60] Lavik EB, Klassen H, Warfvinge K, Langer R, Young MJ. Fabrication of degradable polymer scaffolds to direct the integration and differentiation of retinal progenitors. *Biomaterials*. 2005; 26(16): 3187–3196. 15603813 10.1016/j.biomaterials.2004.08.022

[61] Pritchard CD, Arnér KM, Langer RS, Ghosh FK. Retinal transplantation using surface modified poly(glycerol-co-sebacic acid) membranes. *Biomaterials*. 2010; 31(31): 7978–7984. 20656341 10.1016/j.biomaterials.2010.07.026 PMC4059040

[62] Neeley WL, Redenti S, Klassen H, et al. A microfabricated scaffold for retinal progenitor cell grafting. *Biomaterials*. 2008; 29(4): 418–426. 17961646 10.1016/j.biomaterials.2007.10.007 PMC2174396

[63] Redenti S, Neeley WL, Rompani S, et al. Engineering retinal progenitor cell and scrollable poly(glycerol-sebacate) composites for expansion and subretinal transplantation. *Biomaterials*. 2009; 30(20): 3405–3414. 19361860 10.1016/j.biomaterials.2009.02.046 PMC4109162

[64] Yao J, Ko CW, Baranov PY, et al. Enhanced differentiation and delivery of mouse retinal progenitor cells using a micropatterned biodegradable thin-film polycaprolactone scaffold. *Tissue Eng Part A*. 2015; 21(7-8): 1247–1260. 25517296 10.1089/ten.tea.2013.0720 PMC4394889

[65] Yao J, Tucker BA, Zhang X, Checa-Casalengua P, Herrero-Vanrell R, Young MJ. Robust cell integration from co-transplantation of biodegradable MMP2-PLGA microspheres with retinal progenitor cells. *Biomaterials*. 2011; 32(4): 1041–1050. 21030072 10.1016/j.biomaterials.2010.09.063

[66] Steedman MR, Tao SL, Klassen H, Desai TA. Enhanced differentiation of retinal progenitor cells using microfabricated topographical cues. *Biomed Microdevices*. 2010; 12(3): 363–369. 20077017 10.1007/s10544-009-9392-7 PMC2859162

[67] Ballios BG, Cooke MJ, Donaldson L, et al. A Hyaluronan-Based Injectable Hydrogel Improves the Survival and Integration of Stem Cell Progeny following Transplantation. *Stem Cell Reports*. 2015; 4(6): 1031–1045. 25981414 10.1016/j.stemcr.2015.04.008 PMC4471829

[68] Ballios BG, Cooke MJ, van der Kooy D, Shoichet MS. A hydrogel-based stem cell delivery system to treat retinal degenerative diseases. *Biomaterials*. 2010; 31(9): 2555–2564. 20056272 10.1016/j.biomaterials.2009.12.004

[69] Silverman MS, Hughes SE. Transplantation of photoreceptors to light-damaged retina. *Invest Ophthalmol Vis Sci*. 1989; 30(8): 1684–1690. 2527211

[70] Tomita M, Lavik E, Klassen H, Zahir T, Langer R, Young MJ. Biodegradable polymer composite grafts promote the survival and differentiation of retinal progenitor cells. *Stem cells (Dayton, Ohio)*. 2009; 23(10): 1579–1588. 10.1634/stemcells.2005-0111 16293582

[71] Tao S, Young C, Redenti S, et al. Survival, migration and differentiation of retinal progenitor cells transplanted on micro-machined poly(methyl methacrylate) scaffolds to the subretinal space. *Lab Chip*. 2007; 7(6): 695–701. 17538710 10.1039/b618583e

[72] MacLaren RE, Pearson RA, MacNeil A, et al. Retinal repair by transplantation of photoreceptor precursors. *Nature*. 2006; 444(7116): 203–207. 17093405 10.1038/nature05161

[73] Bartsch U, Oriyakhel W, Kenna PF, et al. Retinal cells integrate into the outer nuclear layer and differentiate into mature photoreceptors after subretinal transplantation into adult mice. *Exp Eye Res*. 2008; 86(4): 691–700. 18329018 10.1016/j.exer.2008.01.018

[74] West EL, Pearson RA, Tschernutter M, Sowden JC, MacLaren RE, Ali RR. Pharmacological disruption of the outer limiting membrane leads to increased retinal integration of transplanted photoreceptor precursors. *Exp Eye Res*. 2008; 86(4): 601–611. 18294631 10.1016/j.exer.2008.01.004 PMC2394572

[75] Pearson RA, Barber AC, West EL, et al. Targeted disruption of outer limiting membrane junctional proteins (Crb1 and ZO-1) increases integration of transplanted photoreceptor precursors into the adult wild-type and degenerating retina. *Cell Transplant*. 2010; 19(4): 487–503. 20089206 10.3727/096368909X486057 PMC2938729

[76] Barber AC, Hippert C, Duran Y, et al. Repair of the degenerate retina by photoreceptor transplantation. *Proc Natl Acad Sci USA*. 2013; 110(1): 354–359. 23248312 10.1073/pnas.1212677110 PMC3538261

[77] Kinouchi R, Takeda M, Yang L, et al. Robust neural integration from retinal transplants in mice deficient in GFAP and vimentin. *Nat Neurosci*. 2003; 6(8): 863–868. 12845328 10.1038/nn1088

[78] Suzuki T, Akimoto M, Imai H, et al. Chondroitinase ABC treatment enhances synaptogenesis between transplant and host neurons in model of retinal degeneration. *Cell Transplant*. 2007; 16(5): 493–503. 17708339 10.3727/000000007783464966

[79] Mandai M, Homma K, Okamoto S, Yamada C, Nomori A, Takahashi M. Adequate Time Window and Environmental Factors Supporting Retinal Graft Cell Survival in RD Mice. *Cell Med*. 2012; 4(1): 45–54. 26858854 10.3727/215517912X639315 PMC4733868

[80] Ma J, Kabiel M, Tucker BA, Ge J, Young MJ. Combining chondroitinase ABC and growth factors promotes the integration of murine retinal progenitor cells transplanted into Rho(-/-) mice. *Mol Vis*. 2011; 17: 1759–1770. 21750603 PMC3133841

[81] Lakowski J, Baron M, Bainbridge J, et al. Cone and rod photoreceptor transplantation in models of the childhood retinopathy Leber congenital amaurosis using flow-sorted Crx-positive donor cells. *Human Molecular Genetics*. 2010; 19(23): 4545–4559. 20858907 10.1093/hmg/ddq378 PMC2972691

[82] Pearson RA, Barber AC, Rizzi M, et al. Restoration of vision after transplantation of photoreceptors. *Nature*. 2012; 485(7396): 99–103. 22522934 10.1038/nature10997 PMC3888831

[83] Smiley S, Nickerson PE, Comanita L, et al. Establishment of a cone photoreceptor transplantation platform based on a novel cone-GFP reporter mouse line. *Sci Rep*. 2016; 6: 22867. 26965927 10.1038/srep22867 PMC4786810

[84] Eberle D, Schubert S, Postel K, Corbeil D, Ader M. Increased integration of transplanted CD73-positive photoreceptor precursors into adult mouse retina. *Invest Ophthalmol Vis Sci*. 2011; 52(9): 6462–6471. 21743009 10.1167/iovs.11-7399

[85] Eberle D, Santos-Ferreira T, Grahl S, Ader M. Subretinal transplantation of MACS purified photoreceptor precursor cells into the adult mouse retina. *J Vis Exp*. 2014;(84): e50932. 10.3791/50932 PMC4130385 24638161

[86] Lakowski J, Han Y-T, Pearson RA, et al. Effective transplantation of photoreceptor precursor cells selected via cell surface antigen expression. *Stem Cells*. 2011; 29(9): 1391–1404. 21774040 10.1002/stem.694 PMC3303132

[87] Jayakody SA, Gonzalez-Cordero A, Ali RR, Pearson RA. Cellular strategies for retinal repair by photoreceptor replacement. *Prog Retin Eye Res*. 2015; 46: 31–66. 25660226 10.1016/j.preteyeres.2015.01.003

[88] Santos-Ferreira T, Postel K, Stutzki H, Kurth T, Zeck G, Ader M. Daylight vision repair by cell transplantation. *Stem Cells*. 2015; 33(1): 79–90. 25183393 10.1002/stem.1824

[89] Singh MS, Charbel Issa P, Butler R, et al. Reversal of end-stage retinal degeneration and restoration of visual function by photoreceptor transplantation. *Proc Natl Acad Sci USA*. 2013; 110(3): 1101–1106. 23288902 10.1073/pnas.1119416110 PMC3549087

[90] Neves J, Zhu J, Sousa-Victor P, et al. Immune modulation by MANF promotes tissue repair and regenerative success in the retina. *Science*. 2016; 353(6294): aaf3646. 27365452 10.1126/science.aaf3646 PMC5270511

[91] West EL, Pearson RA, Barker SE, et al. Long-Term Survival of Photoreceptors Transplanted into the Adult Murine Neural Retina Requires Immune Modulation. *Stem Cells*. 2010; 28(11): 1997–2007. 20857496 10.1002/stem.520 PMC3272388

[92] Singh MS, Aslam SA, Duncan IL, Cramer AO, Barnard AR, MacLaren RE. Cell fusion following photoreceptor transplantation into the non-degenerate retina. *Invest Ophthalmol Vis Sci*. 2014; 55(13): 3989–3989.

[93] Das T, del Cerro M, Jalali S, et al. The transplantation of human fetal neuroretinal cells in advanced retinitis pigmentosa patients: results of a long-term safety study. *Exp Neurol*. 1999; 157(1): 58–68. 10222108 10.1006/exnr.1998.6992

[94] Humayun MS, de Juan E, del Cerro M, et al. Human neural retinal transplantation. *Invest Ophthalmol Vis Sci*. 2000; 41(10): 3100–3106. 10967070

[95] Radtke ND, Aramant RB, Petry HM, Green PT, Pidwell DJ, Seiler MJ. Vision improvement in retinal degeneration patients by implantation of retina together with retinal pigment epithelium. *Am J Ophthalmol*. 2008; 146(2): 172–182. 18547537 10.1016/j.ajo.2008.04.009

[96] Radtke ND, Aramant RB, Seiler MJ, Petry HM, Pidwell D. Vision change after sheet transplant of fetal retina with retinal pigment epithelium to a patient with retinitis pigmentosa. *Arch Ophthalmol*. 2004; 122(8): 1159–1165. 15302656 10.1001/archopht.122.8.1159

[97] Radtke ND, Seiler MJ, Aramant RB, Petry HM, Pidwell DJ. Transplantation of intact sheets of fetal neural retina with its retinal pigment epithelium in retinitis pigmentosa patients. *Am J Ophthalmol*. 2002; 133(4): 544–550. 11931789 10.1016/s0002-9394(02)01322-3

[98] Uyama H, Mandai M, Takahashi M. Stem-cell-based therapies for retinal degenerative diseases: Current challenges in the establishment of new treatment strategies. *Develop Growth Differentiation*. 2021; 63(1): 59–71. 10.1111/dgd.12704 PMC7986097 33315237

[99] Stern JH, Temple S. Stem Cells for Retinal Replacement Therapy. *Neurotherapeutics*. 2011; 8(4): 736–743. 21948217 10.1007/s13311-011-0077-6 PMC3250303

[100] Applications I of M (US) CC on FR and. *Setting the Stage: Fetal Research, Fetal Tissue Research, and Historical Timeline of Regulation and Legislation*. US: National Academies Press; 1994. Accessed June 6, 2021, https://www.ncbi.nlm.nih.gov/books/NBK231997/.

[101] Stem Cell Scientists Answer the Question. Why Is It Important to You to Support Fetal Tissue Research? *Stem Cell Reports*. 2019; 12(2): 186–190. 30759378 10.1016/j.stemcr.2019.01.021 PMC6373626

[102] Schmitt S, Aftab U, Jiang C, et al. Molecular characterization of human retinal progenitor cells. *Invest Ophthalmol Vis Sci*. 2009; 50(12): 5901–5908. 19553622 10.1167/iovs.08-3067

[103] Yang P, Seiler MJ, Aramant RB, Whittemore SR. In vitro isolation and expansion of human retinal progenitor cells. *Exp Neurol*. 2002; 177(1): 326–331. 12429235 10.1006/exnr.2002.7955

[104] Hasan SM, Vugler AA, Miljan EA, Sinden JD, Moss SE, Greenwood J. Immortalized human fetal retinal cells retain progenitor characteristics and represent a potential source for the treatment of retinal degenerative disease. *Cell Transplant*. 2010; 19(10): 1291–1306. 20447347 10.3727/096368910X505477

[105] Wright LS, Pinilla I, Saha J, et al. VSX2 and ASCL1 Are Indicators of Neurogenic Competence in Human Retinal Progenitor Cultures. *PLoS One*. 2015; 10(8): e0135830. 26292211 10.1371/journal.pone.0135830 PMC4546156

[106] Gamm DM, Wright LS, Capowski EE, et al. Regulation of prenatal human retinal neurosphere growth and cell fate potential by retinal pigment epithelium and Mash1. *Stem Cells*. 2008; 26(12): 3182–3193. 18802035 10.1634/stemcells.2008-0300 PMC3127245

[107] Klassen H. Stem cells in clinical trials for treatment of retinal degeneration. *Expert Opin Biol Ther*. 2016; 16(1): 7–14. 26414165 10.1517/14712598.2016.1093110

[108] Klassen H, Kiilgaard JF, Zahir T, et al. Progenitor Cells from the Porcine Neural Retina Express Photoreceptor Markers After Transplantation to the Subretinal Space of Allorecipients. *Stem Cells*. 2007; 25(5): 1222–1230. 17218397 10.1634/stemcells.2006-0541

[109] Semo M, Haamedi N, Stevanato L, et al. Efficacy and Safety of Human Retinal Progenitor Cells. *Transl Vis Sci Technol*. 2016; 5(4): 6. 10.1167/tvst.5.4.6 PMC4959814 27486556

[110] Luo J, Baranov P, Patel S, et al. Human retinal progenitor cell transplantation preserves vision. *J Biol Chem*. 2014; 289(10): 6362–6371. 24407289 10.1074/jbc.M113.513713 PMC3945303

[111] Thomson JA, Itskovitz-Eldor J, Shapiro SS, et al. Embryonic stem cell lines derived from human blastocysts. *Science*. 1998; 282(5391): 1145–1147. 9804556 10.1126/science.282.5391.1145

[112] Klimanskaya I, Hipp J, Rezai KA, West M, Atala A, Lanza R. Derivation and Comparative Assessment of Retinal Pigment Epithelium from Human Embryonic Stem Cells Using Transcriptomics. *Cloning and Stem Cells*. 2004; 6(3): 217–245. 15671670 10.1089/clo.2004.6.217

[113] Ikeda H, Osakada F, Watanabe K, et al. Generation of Rx+/Pax6+ neural retinal precursors from embryonic stem cells. *Proc Natl Acad Sci USA*. 2005; 102(32): 11331–11336. 16076961 10.1073/pnas.0500010102 PMC1183536

[114] Zhao X, Liu J, Ahmad I. Differentiation of embryonic stem cells into retinal neurons. *Biochem Biophys Res Commun*. 2002; 297(2): 177–184. 12237099 10.1016/s0006-291x(02)02126-5

[115] Banin E, Obolensky A, Idelson M, et al. Retinal Incorporation and Differentiation of Neural Precursors Derived from Human Embryonic Stem Cells. *Stem Cells*. 2006; 24(2): 246–257. 16123388 10.1634/stemcells.2005-0009

[116] Lamba DA, Karl MO, Ware CB, Reh TA. Efficient generation of retinal progenitor cells from human embryonic stem cells. *Proc Natl Acad Sci USA*. 2006; 103(34): 12769–12774. 16908856 10.1073/pnas.0601990103 PMC1568922

[117] Osakada F, Ikeda H, Mandai M, et al. Toward the generation of rod and cone photoreceptors from mouse, monkey and human embryonic stem cells. *Nat Biotechnol*. 2008; 26(2): 215–224. 18246062 10.1038/nbt1384

[118] Yu J, Vodyanik MA, Smuga-Otto K, et al. Induced pluripotent stem cell lines derived from human somatic cells. *Science*. 2007; 318(5858): 1917–1920. 18029452 10.1126/science.1151526

[119] Takahashi K, Tanabe K, Ohnuki M, et al. Induction of Pluripotent Stem Cells from Adult Human Fibroblasts by Defined Factors. *Cell*. 2007; 131(5): 861–872. 18035408 10.1016/j.cell.2007.11.019

[120] Pankratz MT, Li X-J, LaVaute TM, Lyons EA, Chen X, Zhang S-C. Directed Neural Differentiation of Human Embryonic Stem Cells via an Obligated Primitive Anterior Stage. *Stem Cells*. 2007; 25(6): 1511–1520. 17332508 10.1634/stemcells.2006-0707 PMC2743478

[121] Hirami Y, Osakada F, Takahashi K, et al. Generation of retinal cells from mouse and human induced pluripotent stem cells. *Neurosci Lett*. 2009; 458(3): 126–131. 19379795 10.1016/j.neulet.2009.04.035

[122] Meyer JS, Shearer RL, Capowski EE, et al. Modeling early retinal development with human embryonic and induced pluripotent stem cells. *Proc Natl Acad Sci USA*. 2009; 106(39): 16698–16703. 19706890 10.1073/pnas.0905245106 PMC2757802

[123] Lamba DA, Gust J, Reh TA. Transplantation of human embryonic stem cell-derived photoreceptors restores some visual function in Crx-deficient mice. *Cell Stem Cell*. 2009; 4(1): 73–79. 19128794 10.1016/j.stem.2008.10.015 PMC2713676

[124] Lamba DA, McUsic A, Hirata RK, Wang PR, Russell D, Reh TA. Generation, purification and transplantation of photoreceptors derived from human induced pluripotent stem cells. *PLoS One*. 2010; 5(1): e8763. 20098701 10.1371/journal.pone.0008763 PMC2808350

[125] Meyer JS, Howden SE, Wallace KA, et al. Optic vesicle-like structures derived from human pluripotent stem cells facilitate a customized approach to retinal disease treatment. *Stem Cells*. 2011; 29(8): 1206–1218. 21678528 10.1002/stem.674 PMC3412675

[126] Nakano T, Ando S, Takata N, et al. Self-Formation of Optic Cups and Storable Stratified Neural Retina from Human ESCs. *Cell Stem Cell*. 2012; 10(6): 771–785. 22704518 10.1016/j.stem.2012.05.009

[127] Zhong X, Gutierrez C, Xue T, et al. Generation of three-dimensional retinal tissue with functional photoreceptors from human iPSCs. *Nat Commun*. 2014; 5: 4047. 24915161 10.1038/ncomms5047 PMC4370190

[128] Kuwahara A, Ozone C, Nakano T, Saito K, Eiraku M, Sasai Y. Generation of a ciliary margin-like stem cell niche from self-organizing human retinal tissue. *Nat Commun*. 2015; 6: 6286. 25695148 10.1038/ncomms7286

[129] Capowski EE, Samimi K, Mayerl SJ, et al. Reproducibility and staging of 3D human retinal organoids across multiple pluripotent stem cell lines. *Development*. 2019; 146(1): dev171686. 30567931 10.1242/dev.171686 PMC6340149

[130] Phillips MJ, Wallace KA, Dickerson SJ, et al. Blood-Derived Human iPS Cells Generate Optic Vesicle–Like Structures with the Capacity to Form Retinal Laminae and Develop Synapses Production of Retina from Human Blood iPS Cells. *Invest Ophthalmol Vis Sci*. 2012; 53(4): 2007–2019. 22410558 10.1167/iovs.11-9313 PMC3648343

[131] Hallam D, Hilgen G, Dorgau B, et al. Human-Induced Pluripotent Stem Cells Generate Light Responsive Retinal Organoids with Variable and Nutrient-Dependent Efficiency. *Stem Cells (Dayton, Ohio)*. 2018; 36(10): 1535–1551. 10.1002/stem.2883 PMC6392112 30004612

[132] Kim S, Lowe A, Dharmat R, et al. Generation, transcriptome profiling, and functional validation of cone-rich human retinal organoids. *Proc Natl Acad Sci USA*. 2019; 116(22): 10824–10833. 31072937 10.1073/pnas.1901572116 PMC6561190

[133] Bell CM, Zack DJ, Berlinicke CA. Human Organoids for the Study of Retinal Development and Disease. *Annu Rev Vis Sci*. 2020; 6: 91–114. 32936736 10.1146/annurev-vision-121219-081855

[134] Cowan CS, Renner M, De Gennaro M, et al. Cell Types of the Human Retina and Its Organoids at Single-Cell Resolution. *Cell*. 2020; 182(6): 1623–1640.e34. 32946783 10.1016/j.cell.2020.08.013 PMC7505495

[135] Phillips MJ, Jiang P, Howden S, et al. A Novel Approach to Single Cell RNA-Sequence Analysis Facilitates In Silico Gene Reporting of Human Pluripotent Stem Cell-Derived Retinal Cell Types. *Stem Cells*. 2018; 36(3): 313–324. 29230913 10.1002/stem.2755 PMC5823737

[136] Assawachananont J, Mandai M, Okamoto S, et al. Transplantation of embryonic and induced pluripotent stem cell-derived 3D retinal sheets into retinal degenerative mice. *Stem Cell Reports*. 2014; 2(5): 662–674. 24936453 10.1016/j.stemcr.2014.03.011 PMC4050483

[137] Decembrini S, Koch U, Radtke F, Moulin A, Arsenijevic Y. Derivation of Traceable and Transplantable Photoreceptors from Mouse Embryonic Stem Cells. *Stem Cell Reports*. 2014; 2(6): 853–865. 24936471 10.1016/j.stemcr.2014.04.010 PMC4050344

[138] Gonzalez-Cordero A, West EL, Pearson RA, et al. Photoreceptor precursors derived from three-dimensional embryonic stem cell cultures integrate and mature within adult degenerate retina. *Nat Biotechnol*. 2013; 31(8): 741–747. 23873086 10.1038/nbt.2643 PMC3826328

[139] Kruczek K, Gonzalez-Cordero A, Goh D, et al. Differentiation and Transplantation of Embryonic Stem Cell-Derived Cone Photoreceptors into a Mouse Model of End-Stage Retinal Degeneration. *Stem Cell Reports*. 2017; 8(6): 1659–1674. 28552606 10.1016/j.stemcr.2017.04.030 PMC5470175

[140] Santos-Ferreira T, Völkner M, Borsch O, et al. Stem Cell–Derived Photoreceptor Transplants Differentially Integrate Into Mouse Models of Cone-Rod Dystrophy. *Invest Ophthalmol Vis Sci*. 2016; 57(7): 3509–3520. 27367586 10.1167/iovs.16-19087

[141] Mandai M, Fujii M, Hashiguchi T, et al. iPSC-Derived Retina Transplants Improve Vision in rd1 End-Stage Retinal-Degeneration Mice. *Stem Cell Reports*. 2017; 8(1): 69–83. 28076757 10.1016/j.stemcr.2016.12.008 PMC5233464

[142] Tucker BA, Park I-H, Qi SD, et al. Transplantation of Adult Mouse iPS Cell-Derived Photoreceptor Precursors Restores Retinal Structure and Function in Degenerative Mice. *PLoS One*. 2011; 6(4): e18992. 21559507 10.1371/journal.pone.0018992 PMC3084746

[143] West EL, Gonzalez-Cordero A, Hippert C, et al. Defining the integration capacity of embryonic stem cell-derived photoreceptor precursors. *Stem Cells*. 2012; 30(7): 1424–1435. 22570183 10.1002/stem.1123 PMC3580313

[144] Gonzalez-Cordero A, Kruczek K, Naeem A, et al. Recapitulation of Human Retinal Development from Human Pluripotent Stem Cells Generates Transplantable Populations of Cone Photoreceptors. *Stem Cell Reports*. 2017; 9(3): 820–837. 28844659 10.1016/j.stemcr.2017.07.022 PMC5599247

[145] Zhu J, Cifuentes H, Reynolds J, Lamba DA. Immunosuppression via Loss of IL2rγ Enhances Long-Term Functional Integration of hESC-Derived Photoreceptors in the Mouse Retina. *Cell Stem Cell*. 2017; 20(3): 374–384.e5. 28089909 10.1016/j.stem.2016.11.019

[146] Iraha S, Tu H-Y, Yamasaki S, et al. Establishment of Immunodeficient Retinal Degeneration Model Mice and Functional Maturation of Human ESC-Derived Retinal Sheets after Transplantation. *Stem Cell Reports*. 2018; 10(3): 1059–1074. 29503091 10.1016/j.stemcr.2018.01.032 PMC5918611

[147] Tu H-Y, Watanabe T, Shirai H, et al. Medium- to long-term survival and functional examination of human iPSC-derived retinas in rat and primate models of retinal degeneration. *EBioMedicine*. 2019; 39: 562–574. 30502055 10.1016/j.ebiom.2018.11.028 PMC6354559

[148] Shirai H, Mandai M, Matsushita K, et al. Transplantation of human embryonic stem cell-derived retinal tissue in two primate models of retinal degeneration. *Proc Natl Acad Sci U S A*. 2016; 113(1): E81–E90. 26699487 10.1073/pnas.1512590113 PMC4711854

[149] McLelland BT, Lin B, Mathur A, et al. Transplanted hESC-Derived Retina Organoid Sheets Differentiate, Integrate, and Improve Visual Function in Retinal Degenerate Rats. *Invest Ophthalmol Vis Sci*. 2018; 59(6): 2586–2603. 29847666 10.1167/iovs.17-23646 PMC5968836

[150] Seiler MJ, Aramant RB, Jones MK, Ferguson DL, Bryda EC, Keirstead HS. A new immunodeficient pigmented retinal degenerate rat strain to study transplantation of human cells without immunosuppression. *Graefes Arch Clin Exp Ophthalmol*. 2014; 252(7): 1079–1092. 24817311 10.1007/s00417-014-2638-y PMC4374984

[151] Hambright D, Park KY, Brooks M, McKay R, Swaroop A, Nasonkin IO. Long-term survival and differentiation of retinal neurons derived from human embryonic stem cell lines in un-immunosuppressed mouse retina. *Molecular Vision*. 2012; 18: 920–936. 22539871 PMC3335781

[152] Singh MS, Balmer J, Barnard AR, et al. Transplanted photoreceptor precursors transfer proteins to host photoreceptors by a mechanism of cytoplasmic fusion. *Nat Commun*. 2016; 7: 13537. 27901042 10.1038/ncomms13537 PMC5141374

[153] Pearson RA, Gonzalez-Cordero A, West EL, et al. Donor and host photoreceptors engage in material transfer following transplantation of post-mitotic photoreceptor precursors. *Nat Commun*. 2016; 7: 13029. 27701378 10.1038/ncomms13029 PMC5059468

[154] Santos-Ferreira T, Llonch S, Borsch O, Postel K, Haas J, Ader M. Retinal transplantation of photoreceptors results in donor-host cytoplasmic exchange. *Nat Commun*. 2016; 7: 13028. 27701381 10.1038/ncomms13028 PMC5059459

[155] Ortin-Martinez A, Tsai ELS, Nickerson PE, et al. A Reinterpretation of Cell Transplantation: GFP Transfer From Donor to Host Photoreceptors. *Stem Cells*. 2017; 35(4): 932–939. 27977075 10.1002/stem.2552

[156] Decembrini S, Martin C, Sennlaub F, et al. Cone Genesis Tracing by the Chrnb4-EGFP Mouse Line: Evidences of Cellular Material Fusion after Cone Precursor Transplantation. *Mol Ther*. 2017; 25(3): 634–653. 28143742 10.1016/j.ymthe.2016.12.015 PMC5363218

[157] Waldron PV, Di Marco F, Kruczek K, et al. Transplanted Donor- or Stem Cell-Derived Cone Photoreceptors Can Both Integrate and Undergo Material Transfer in an Environment-Dependent Manner. *Stem Cell Reports*. 2018; 10(2): 406–421. 29307580 10.1016/j.stemcr.2017.12.008 PMC5830910

[158] MacLaren RE. Cone fusion confusion in photoreceptor transplantation. *Stem Cell Investig*. 2017; 4: 71. 10.21037/sci.2017.08.02 PMC5590021 28920064

[159] Barnea-Cramer AO, Wang W, Lu S-J, et al. Function of human pluripotent stem cell-derived photoreceptor progenitors in blind mice. *Sci Rep*. 2016; 6: 29784. 27405580 10.1038/srep29784 PMC4942817

[160] Aboualizadeh E, Phillips MJ, McGregor JE, et al. Imaging Transplanted Photoreceptors in Living Nonhuman Primates with Single-Cell Resolution. *Stem Cell Reports*. 2020; 15(2): 482–497. 32707075 10.1016/j.stemcr.2020.06.019 PMC7419740

[161] Ribeiro J, Procyk CA, West EL, et al. Restoration of visual function in advanced disease after transplantation of purified human pluripotent stem cell-derived cone photoreceptors. *Cell Reports*. 2021; 35(3): 109022. 33882303 10.1016/j.celrep.2021.109022 PMC8065177

[162] Gagliardi G, Ben M’Barek K, Chaffiol A, et al. Characterization and Transplantation of CD73-Positive Photoreceptors Isolated from Human iPSC-Derived Retinal Organoids. *Stem Cell Reports*. 2018; 11(3): 665–680. 30100409 10.1016/j.stemcr.2018.07.005 PMC6135113

[163] Garita-Hernandez M, Lampič M, Chaffiol A, et al. Restoration of visual function by transplantation of optogenetically engineered photoreceptors. *Nature Communications*. 2019; 10(1): 4524. 10.1038/s41467-019-12330-2 PMC6778196 31586094

[164] Wiley LA, Burnight ER, DeLuca AP, et al. cGMP production of patient-specific iPSCs and photoreceptor precursor cells to treat retinal degenerative blindness. *Sci Rep*. 2016; 6: 30742. 27471043 10.1038/srep30742 PMC4965859

[165] Reichman S, Slembrouck A, Gagliardi G, et al. Generation of Storable Retinal Organoids and Retinal Pigmented Epithelium from Adherent Human iPS Cells in Xeno-Free and Feeder-Free Conditions. *Stem Cells (Dayton, Ohio)*. 2017; 35(5): 1176–1188. 10.1002/stem.2586 28220575

[166] Stone NE, Voigt AP, Mullins RF, Sulchek T, Tucker BA. Microfluidic processing of stem cells for autologous cell replacement. *Stem Cells Transl Med*. 2021; 10(10): 1384–1393. 34156760 10.1002/sctm.21-0080 PMC8459636

[167] Huang C-Y, Liu C-L, Ting C-Y, et al. Human iPSC banking: barriers and opportunities. *J Biomed Sci*. 2019; 26: 87. 31660969 10.1186/s12929-019-0578-x PMC6819403

[168] Kramer J, Chirco KR, Lamba DA. Immunological Considerations for Retinal Stem Cell Therapy. In: Bharti K, ed. *Pluripotent Stem Cells in Eye Disease Therapy*. Advances in Experimental Medicine and Biology. New York, NY: Springer International Publishing; 2019: 99–119. 10.1007/978-3-030-28471-8_4 31654387

[169] Petrus-Reurer S, Winblad N, Kumar P, et al. Generation of Retinal Pigment Epithelial Cells Derived from Human Embryonic Stem Cells Lacking Human Leukocyte Antigen Class I and II. *Stem Cell Reports*. 2020; 14(4): 648–662. 32197113 10.1016/j.stemcr.2020.02.006 PMC7160308

[170] Creasey AA, Stacey G, Bharti K, Sato Y, Lubiniecki A. A strategic road map to filing a Biologics License Application for a pluripotent stem cell derived therapeutic product. *Biologicals*. 2019; 59: 68–71. 31053443 10.1016/j.biologicals.2019.03.007 PMC7476766

[171] Wright LS, Phillips MJ, Pinilla I, Hei D, Gamm DM. Induced pluripotent stem cells as custom therapeutics for retinal repair: Progress and rationale. *Exp Eye Res*. 2014; 123: 161–172. 24534198 10.1016/j.exer.2013.12.001 PMC4047146

[172] Ovando-Roche P, West EL, Branch MJ, et al. Use of bioreactors for culturing human retinal organoids improves photoreceptor yields. *Stem Cell Res Ther*. 2018; 9(1): 156. 29895313 10.1186/s13287-018-0907-0 PMC5998504

[173] Xue Y, Seiler MJ, Tang WC, et al. Retinal organoids on-a-chip: a micro-millifluidic bioreactor for long-term organoid maintenance. *Lab Chip*. 2021; 21(17): 3361–3377. 34236056 10.1039/d1lc00011j PMC8387452

[174] Koso H, Minami C, Tabata Y, et al. CD73, a novel cell surface antigen that characterizes retinal photoreceptor precursor cells. *Invest Ophthalmol Vis Sci*. 2009; 50(11): 5411–5418. 19515998 10.1167/iovs.08-3246

[175] Stone NE, Voigt AP, Cooke JA, et al. Label-free microfluidic enrichment of photoreceptor cells. *Exp Eye Res*. 2020; 199: 108166. 32771499 10.1016/j.exer.2020.108166 PMC12646284

[176] Lakowski J, Welby E, Budinger D, et al. Isolation of Human Photoreceptor Precursors via a Cell Surface Marker Panel from Stem Cell-Derived Retinal Organoids and Fetal Retinae. *Stem Cells*. 2018; 36(5): 709–722. 29327488 10.1002/stem.2775 PMC5947711

[177] Collin J, Zerti D, Queen R, et al. CRX Expression in Pluripotent Stem Cell-Derived Photoreceptors Marks a Transplantable Subpopulation of Early Cones. *Stem Cells*. 2019; 37(5): 609–622. 30681766 10.1002/stem.2974 PMC6519156

[178] Ludwig A, Phillips J, Jager L, et al. Transplantation of human pluripotent stem cell-derived photoreceptors on a biocompatible scaffold in the S334ter rat. *Invest Ophthalmol Vis Sci*. 2019; 60(9): 2886–2886.

[179] Lin B, McLelland BT, Aramant RB, et al. Retina Organoid Transplants Develop Photoreceptors and Improve Visual Function in RCS Rats With RPE Dysfunction. *Invest Ophthalmol Vis Sci*. 2020; 61(11): 34. 10.1167/iovs.61.11.34 PMC7509771 32945842

[180] Phillips MJ, Capowski EE, Petersen A, et al. Generation of a rod-specific NRL reporter line in human pluripotent stem cells. *Scientific Reports*. 2018; 8(1): 2370. 29402929 10.1038/s41598-018-20813-3 PMC5799252

[181] Kaewkhaw R, Swaroop M, Homma K, et al. Treatment Paradigms for Retinal and Macular Diseases Using 3-D Retina Cultures Derived From Human Reporter Pluripotent Stem Cell Lines. *Invest Ophthalmol Vis Sci*. 2016; 57(5): ORSFl1–ORSFl11. 27116668 10.1167/iovs.15-17639 PMC4855830

[182] Kaewkhaw R, Kaya KD, Brooks M, et al. Transcriptome Dynamics of Developing Photoreceptors in Three-Dimensional Retina Cultures Recapitulates Temporal Sequence of Human Cone and Rod Differentiation Revealing Cell Surface Markers and Gene Networks. *Stem Cells (Dayton, Ohio)*. 2015; 33(12): 3504–3518. 10.1002/stem.2122 PMC4713319 26235913

[183] Vergara MN, Flores-Bellver M, Aparicio-Domingo S, et al. Three-dimensional automated reporter quantification (3D-ARQ) technology enables quantitative screening in retinal organoids. *Development*. 2017; 144(20): 3698–3705. 28870990 10.1242/dev.146290 PMC5675442

[184] McClements ME, Staurenghi F, MacLaren RE, Cehajic-Kapetanovic J. Optogenetic Gene Therapy for the Degenerate Retina: Recent Advances. *Front Neurosci*. 2020; 14: 570909. 33262683 10.3389/fnins.2020.570909 PMC7686539

[185] Singh R, Phillips MJ, Kuai D, et al. Functional analysis of serially expanded human iPS cell-derived RPE cultures. *Invest Ophthalmol Vis Sci*. 2013; 54(10): 6767–6778. 24030465 10.1167/iovs.13-11943 PMC3799561

[186] Sinha D, Steyer B, Shahi PK, et al. Human iPSC Modeling Reveals Mutation-Specific Responses to Gene Therapy in a Genotypically Diverse Dominant Maculopathy. *Am J Hum Genet*. 2020; 107(2): 278–292. 32707085 10.1016/j.ajhg.2020.06.011 PMC7413860

[187] Engle SJ, Blaha L, Kleiman RJ. Best Practices for Translational Disease Modeling Using Human iPSC-Derived Neurons. *Neuron*. 2018; 100(4): 783–797. 30465765 10.1016/j.neuron.2018.10.033

[188] Kruczek K, Swaroop A. Pluripotent stem cell-derived retinal organoids for disease modeling and development of therapies. *Stem Cells*. 2020; 38: 1206–1215. 32506758 10.1002/stem.3239 PMC7586922

[189] Garita-Hernandez M, Chaffiol A, Guibbal L, et al. Control of Microbial Opsin Expression in Stem Cell Derived Cones for Improved Outcomes in Cell Therapy. *Front Cell Neurosci*. 2021; 15: 648210. 33815066 10.3389/fncel.2021.648210 PMC8012682

[190] Akiba R, Matsuyama T, Tu HY, et al. Quantitative and Qualitative Evaluation of Photoreceptor Synapses in Developing, Degenerating and Regenerating Retinas. *Frontiers in Cellular Neuroscience*. 2019; 13: 16. 30804754 10.3389/fncel.2019.00016 PMC6378395

[191] Worthington KS, Wiley LA, Kaalberg EE, et al. Two-photon polymerization for production of human iPSC-derived retinal cell grafts. *Acta Biomater*. 2017; 55: 385–395. 28351682 10.1016/j.actbio.2017.03.039 PMC5495181

[192] Jung YH, Phillips MJ, Lee J, et al. 3D Microstructured Scaffolds to Support Photoreceptor Polarization and Maturation. *Advanced Materials (Deerfield Beach, Fla)*. 2018; 30(39): e1803550. 10.1002/adma.201803550 30109736

[193] Lee I-K, Ludwig AL, Phillips MJ, et al. Ultrathin micromolded 3D scaffolds for high-density photoreceptor layer reconstruction. *Science Advances*. 2021; 7(17): eabf0344. 33883135 10.1126/sciadv.abf0344 PMC8059936

[194] Sharma R, Khristov V, Rising A, et al. Clinical-grade stem cell-derived retinal pigment epithelium patch rescues retinal degeneration in rodents and pigs. *Sci Transl Med*. 2019; 11(475): eaat5580. 30651323 10.1126/scitranslmed.aat5580 PMC8784963

[195] Kashani AH, Uang J, Mert M, et al. Surgical Method for Implantation of a Biosynthetic Retinal Pigment Epithelium Monolayer for Geographic Atrophy: Experience from a Phase 1/2a Study. *Ophthalmol Retina*. 2020; 4: 264–273. 31786135 10.1016/j.oret.2019.09.017

[196] Thompson JR, Worthington KS, Green BJ, et al. Two-photon polymerized poly(caprolactone) retinal cell delivery scaffolds and their systemic and retinal biocompatibility. *Acta Biomaterialia*. 2019; 941: 204–218. 10.1016/j.actbio.2019.04.057 PMC6659122 31055121

[197] Wendland RJ, Jiao C, Russell SR, et al. The effect of retinal scaffold modulus on performance during surgical handling. *Exp Eye Res*. 2021; 207: 108566. 33838142 10.1016/j.exer.2021.108566 PMC8187337

[198] Singh MS, MacLaren RE. Stem Cell Treatment for Age-Related Macular Degeneration: the Challenges. *Invest Ophthalmol Vis Sci*. 2018; 59(4): AMD78–AMD82. 30025109 10.1167/iovs.18-24426

[199] Klassen H, Kiilgaard JF, Warfvinge K, et al. Photoreceptor Differentiation following Transplantation of Allogeneic Retinal Progenitor Cells to the Dystrophic Rhodopsin Pro347Leu Transgenic Pig. *Stem Cells International*. 2012; 2012: e939801. 10.1155/2012/939801 PMC3337587 22567027

[200] Zhao X, van Praag H. Steps towards standardized quantification of adult neurogenesis. *Nature Communications*. 2020; 11(1): 4275. 10.1038/s41467-020-18046-y PMC7450090 32848155

[201] Schmitz C, Hof PR. Design-based stereology in neuroscience. *Neuroscience*. 2005; 130(4): 813–831. 15652981 10.1016/j.neuroscience.2004.08.050

[202] Schmitz C, Eastwood BS, Tappan SJ, Glaser JR, Peterson DA, Hof PR. Current automated 3D cell detection methods are not a suitable replacement for manual stereologic cell counting. *Front Neuroanat*. 2014; 8: 27. 24847213 10.3389/fnana.2014.00027 PMC4019880

[203] Zerti D, Hilgen G, Dorgau B, et al. Transplanted pluripotent stem cell-derived photoreceptor precursors elicit conventional and unusual light responses in mice with advanced retinal degeneration. *Stem Cells*. 2021; 39: 882–896. 33657251 10.1002/stem.3365

[204] Zhang KY, Aguzzi EA, Johnson TV. Retinal Ganglion Cell Transplantation: Approaches for Overcoming Challenges to Functional Integration. *Cells*. 2021; 10(6): 1426. 34200991 10.3390/cells10061426 PMC8228580

[205] Zerti D, Hilgen G, Dorgau B, et al. Transplanted pluripotent stem cell-derived photoreceptor precursors elicit conventional and unusual light responses in mice with advanced retinal degeneration. *Stem Cells*. 2021; 39: 882–896. 33657251 10.1002/stem.3365

[206] Laver CRJ, Matsubara JA. Structural divergence of essential triad ribbon synapse proteins among placental mammals – Implications for preclinical trials in photoreceptor transplantation therapy. *Experimental Eye Research*. 2017; 159: 156–167. 28322827 10.1016/j.exer.2017.03.005

[207] Pardue MT, Phillips MJ, Yin H, et al. Neuroprotective Effect of Subretinal Implants in the RCS Rat. *Invest Ophthalmol Vis Sci*. 2005; 46(2): 674–682. 15671299 10.1167/iovs.04-0515

[208] Bryda EC, LaVail MM. Letter to the editor announcing the availability of RCS and transgenic rats with P23H and S334ter rhodopsin mutations with inherited retinal degenerations. *Exp Eye Res*. 2019; 178: 176. 30296413 10.1016/j.exer.2018.10.003

[209] Fujii M, Sunagawa GA, Kondo M, Takahashi M, Mandai M. Evaluation of micro Electroretinograms Recorded with Multiple Electrode Array to Assess Focal Retinal Function. *Sci Rep*. 2016; 6(1): 30719. 27480484 10.1038/srep30719 PMC4969741

[210] Lyubarsky A, Bennett J. Light Avoidance in Mice May Be not Related to Photoreceptor-Driven Input. *Invest Ophthalmol Vis Sci*. 2016; 57(12): 2768–2768.

[211] Winkler PA, Occelli LM, Petersen-Jones SM. Large Animal Models of Inherited Retinal Degenerations: A Review. *Cells*. 2020; 9(4): 882. 10.3390/cells9040882 PMC7226744 32260251

[212] Lamb TD. Evolution of vertebrate retinal photoreception. *Philosophical Transactions of the Royal Society B: Biological Sciences*. 2009; 364(1531): 2911–2924. 10.1098/rstb.2009.0102 PMC2781864 19720653

[213] Zarbin M. Cell-Based Therapy for Degenerative Retinal Disease. *Trends in Molecular Medicine*. 2016; 22(2): 115–134. 26791247 10.1016/j.molmed.2015.12.007

[214] Zarbin M. Cell-Based Therapy for Retinal Disease: The New Frontier. *Methods Mol Biol*. 2019; 1834: 367–381. 30324455 10.1007/978-1-4939-8669-9_23

[215] Wynne N, Carroll J, Duncan JL. Promises and pitfalls of evaluating photoreceptor-based retinal disease with adaptive optics scanning light ophthalmoscopy (AOSLO). *Progress in Retinal and Eye Research*. 2021; 83: 100920. 33161127 10.1016/j.preteyeres.2020.100920 PMC8639282

[216] McGregor JE, Williams DR, Merigan WH. Functional Assessment of Vision Restoration. *Adv Exp Med Biol*. 2019; 1185: 145–149. 31884603 10.1007/978-3-030-27378-1_24

[217] Thompson DA, Iannaccone A, Ali RR, et al. Advancing Clinical Trials for Inherited Retinal Diseases: Recommendations from the Second Monaciano Symposium. *Transl Vis Sci Technol*. 2020; 9(7): 2. 10.1167/tvst.9.7.2 PMC7414644 32832209

[218] Liu YV, Sodhi SK, Xue G, et al. Quantifiable In Vivo Imaging Biomarkers of Retinal Regeneration by Photoreceptor Cell Transplantation. *Trans Vis Sci Tech*. 2020; 9(7): 5. 10.1167/tvst.9.7.5 PMC7414711 32832212

[219] Reh TA. Photoreceptor Transplantation in Late Stage Retinal Degeneration. *Invest Ophthalmol Vis Sci*. 2016; 57(5): ORSFg1–7. 27116664 10.1167/iovs.15-17659 PMC5992960

[220] Hippert C, Graca AB, Basche M, et al. RNAi-mediated suppression of vimentin or glial fibrillary acidic protein prevents the establishment of Müller glial cell hypertrophy in progressive retinal degeneration. *Glia*. 2021; 69: 2272–2290. 34029407 10.1002/glia.24034

[221] Chan K, Hoon M, Pattnaik BR, et al. Vigabatrin-Induced Retinal Functional Alterations and Second-Order Neuron Plasticity in C57BL/6J Mice. *Invest Ophthalmol Vis Sci*. 2020; 61(2): 17. 10.1167/iovs.61.2.17 PMC7326505 32053727

[222] Tsai ELS, Ortin-Martinez A, Gurdita A, et al. Modeling of Photoreceptor Donor-Host Interaction Following Transplantation Reveals a Role for Crx, Müller Glia, and Rho/ROCK Signaling in Neurite Outgrowth. *Stem Cells*. 2019; 37(4): 529–541. 30715780 10.1002/stem.2985

[223] Hoon M, Okawa H, Della Santina L, Wong ROL. Functional architecture of the retina: development and disease. *Prog Retin Eye Res*. 2014; 42: 44–84. 24984227 10.1016/j.preteyeres.2014.06.003 PMC4134977

[224] Itoh M, Mukae Y, Kitsuka T, et al. Development of an immunodeficient pig model allowing long-term accommodation of artificial human vascular tubes. *Nature Communications*. 2019; 10(1): 2244. 10.1038/s41467-019-10107-1 PMC6529409 31113942

[225] Singh RK, Occelli LM, Binette F, Petersen-Jones SM, Nasonkin IO. Transplantation of Human Embryonic Stem Cell-Derived Retinal Tissue in the Subretinal Space of the Cat Eye. *Stem Cells and Development*. 2019; 28(17): 1151–1166. 31210100 10.1089/scd.2019.0090 PMC6708274

[226] Yamasaki S, Sugita S, Horiuchi M, et al. Low Immunogenicity and Immunosuppressive Properties of Human ESC- and iPSC-Derived Retinas. *Stem Cell Reports*. 2021; 16(4): 851–867. 33770500 10.1016/j.stemcr.2021.02.021 PMC8072071

[227] Saraf Steven S, Cunningham Matthew A, Kuriyan Ajay E, et al. Bilateral Retinal Detachments After Intravitreal Injection of Adipose-Derived ‘Stem Cells’ in a Patient With Exudative Macular Degeneration. *Ophthalmic Surgery, Lasers and Imaging Retina*. 2017; 48(9): 772–775. 10.3928/23258160-20170829-16 28902341

[228] Leung Ella H, Flynn Harry W, Albini Thomas A, Medina Carlos A. Retinal Detachment After Subretinal Stem Cell Transplantation. *Ophthalmic Surgery, Lasers and Imaging Retina*. 2016; 47(6): 600–601. 10.3928/23258160-20160601-16 27327294

[229] Kuriyan AE, Albini TA, Townsend JH, et al. Vision Loss after Intravitreal Injection of Autologous “Stem Cells” for AMD. *N Engl J Med*. 2017; 376: 1047–1053. 28296617 10.1056/NEJMoa1609583 PMC5551890

[230] Knoepfler PS. The Stem Cell Hard Sell: Report from a Clinic's Patient Recruitment Seminar. *Stem Cells Translational Medicine*. 2017; 6(1): 14–16. 28170181 10.5966/sctm.2016-0208 PMC5442738

[231] Cossu G, Birchall M, Brown T, et al. Lancet Commission: Stem cells and regenerative medicine. *The Lancet*. 2018; 391(10123): 883–910. 10.1016/S0140-6736(17)31366-1 28987452

[232] Patient Resources. A Closer Look at Stem Cells. Accessed May 27, 2021, https://www.closerlookatstemcells.org/patient-resources/.

[233] Age-Related Macular Degeneration. A Closer Look at Stem Cells. Accessed May 27, 2021, https://www.closerlookatstemcells.org/stem-cells-medicine/age-related-macular-degeneration/.

[234] Zarbin M. What Constitutes Translational Research? Implications for the Scope of Translational Vision Science and Technology. *Transl Vis Sci Technol*. 2020; 9(8): 22. 10.1167/tvst.9.8.22 PMC7422760 32855869

[235] Haeckel EHPA. *Anthropogenie*. Germany: W. Engelmann; 1877.

[236] Boveri: Ueber die Befruchtung der Eier von Ascaris... - Google Scholar. Accessed September 9, 2021, https://scholar.google.com/scholar_lookup?title=%C3%9Cber%20die%20Befruchtung%20der%20Eier%20von%20Ascaris%20megalocephala&author=T.%20Boveri&journal=Sitzungsberichte%20der%20Gesellschaft%20f%C3%BCr%20Morphologie%20und%20Physiologie%20in%20M%C3%BCnchen&volume=3&pages=71-80&publication_year=1887.

[237] Häcker V. Die Kerntheilungsvorgänge bei der Mesodermund Entodermbildung von Cyclops. *Archiv f mikrosk Anatomie*. 1892; 39(1): 556–581.

[238] Thomas ED, Lochte HL, Lu WC, Ferrebee JW. Intravenous Infusion of Bone Marrow in Patients Receiving Radiation and Chemotherapy. *N Engl J Med*. 1957; 257(11): 491–496. 13464965 10.1056/NEJM195709122571102

[239] Till JE, McCulloch EA. A Direct Measurement of the Radiation Sensitivity of Normal Mouse Bone Marrow Cells. *Radiation Research*. 1961; 14(2): 213–222. 13776896

[240] Gurdon JB. The developmental capacity of nuclei taken from intestinal epithelium cells of feeding tadpoles. *J Embryol Exp Morphol*. 1962; 10: 622–640. 13951335

[241] Evans MJ, Kaufman MH. Establishment in culture of pluripotential cells from mouse embryos. *Nature*. 1981; 292(5819): 154–156. 7242681 10.1038/292154a0

[242] Martin GR. Isolation of a pluripotent cell line from early mouse embryos cultured in medium conditioned by teratocarcinoma stem cells. *Proc Natl Acad Sci USA*. 1981; 78(12): 7634–7638. 6950406 10.1073/pnas.78.12.7634 PMC349323

[243] Thomson JA, Kalishman J, Golos TG, et al. Isolation of a primate embryonic stem cell line. *Proc Natl Acad Sci USA*. 1995; 92(17): 7844–7848. 7544005 10.1073/pnas.92.17.7844 PMC41242

[244] Wilmut I, Schnieke AE, McWhir J, Kind AJ, Campbell KH. Viable offspring derived from fetal and adult mammalian cells. *Nature*. 1997; 385(6619): 810–813. 9039911 10.1038/385810a0

[245] Takahashi K, Yamanaka S. Induction of pluripotent stem cells from mouse embryonic and adult fibroblast cultures by defined factors. *Cell*. 2006; 126(4): 663–676. 16904174 10.1016/j.cell.2006.07.024

[246] Kondo M, Sakai T, Komeima K, et al. Generation of a transgenic rabbit model of retinal degeneration. *Invest Ophthalmol Vis Sci*. 2009; 50(3): 1371–1377. 19074802 10.1167/iovs.08-2863

[247] Wang Z, Feng C, Yang R, et al. Large-Area Photoreceptor Degeneration Model in Rabbits by Photocoagulation and Oxidative Stress in the Retina. *Frontiers in Neuroscience*. 2021; 15: 640. 10.3389/fnins.2021.617175 PMC8222581 34177442

[248] Waide EH, Dekkers JCM, Ross JW, et al. Not All SCID Pigs Are Created Equally: Two Independent Mutations in the Artemis Gene Cause SCID in Pigs. *J Immunol*. 2015; 195(7): 3171–3179. 26320255 10.4049/jimmunol.1501132 PMC5621739

[249] Stanzel B, Ader M, Liu Z, et al. Surgical Approaches for Cell Therapeutics Delivery to the Retinal Pigment Epithelium and Retina. In: Bharti K, ed. *Pluripotent Stem Cells in Eye Disease Therapy*. Advances in Experimental Medicine and Biology. New York, NY: Springer International Publishing; 2019: 141–170. 10.1007/978-3-030-28471-8_6 31654389

