# Supplementary material for: Outer Retinal Cell Replacement: Putting the Pieces Together
Source: Transl Vis Sci Technol. 2021 Nov 1;10(10):15. doi: 10.1167/tvst.10.10.15 (PMC8572485; doi:10.1167/tvst.10.10.15)
Supplement: Supplement 1 [file tvst-10-10-15_s001.pdf]

Supplementary Material

Outer retinal cell replacement: Putting the pieces together

Allison L. Ludwig[1-3], David M. Gamm[1-3]

[1] Waisman Center, University of Wisconsin–Madison, Madison, WI 53705

[2] McPherson Eye Research Institute, University of Wisconsin–Madison, Madison, WI 53705

[3] Department of Ophthalmology and Visual Sciences, University of Wisconsin–Madison, Madison, WI 53705

Supplemental Note 1: Defining integration in retinal cell replacement.

Although the term has appeared in the retinal regeneration literature across its entire history[1,2], the exact definition of integration is a present topic of debate within the field. A non-integrated donor cell—generally found in a self-contained mass in the subretinal space (SRS), floating in the vitreous, or walled off by glial scarring[3]—is more easily defined than the integrated cell. Since many have historically relied on fluorescent reporters to identify donor cells, most studies prior to the mid-2010s referred to any fluorescent donor cells found within the SRS as "incorporated" or "integrated". The discovery of donor-to-host cytoplasmic transfer of fluorophores has highlighted the need for stringent, universal criteria to distinguish true integration from material exchange events (see Nickerson *et al.,* 2018[4] for further discussion). The field has since adopted a two-layered approach to identify and define integration[4]. Apparent donor PRP within the SRS should ideally: 1) express cytoplasmic markers specific to the donor cell population (e.g. *CRX-TdTomato*, *L/Mopsin-GFP,* Recoverin, etc.) <u>and</u> 2) co-label with at least one nuclear donor-specific marker (e.g., human nuclear antigen [HNA], Y-chromosome fluorescent in situ hybridization [FISH], BrdU, etc.). Nuclear diameter[2,5] and cellular morphology[6] are also useful adjunct approaches for distinguishing donor PRP from host cells.

Distinguishing between *anatomic integration* (i.e., histologic or ultrastructural evidence of synapse formation) and *functional integration* (i.e., direct evidence of donor cell-initiated synaptic transmission) is also critical. Recent computational modeling suggests that anatomic integration may not directly correlate with functional integration or visual behavior in xenotransplants due to evolutionarily divergent features in triad ribbon synapses[7] and visual function[8] in mammals. Many studies referenced in the present review have assessed *anatomic integration* and its association with light-evoked behavior. However, current studies aimed at exploring *functional integration*—often with electrophysiologic and/or behavioral assessments—often assay several synapses downstream of the donor-to-host PR synapse, making it difficult to control for alternate mechanisms of functional recovery. Methods to directly assay *functional integration* at the level of PR donor-to-host synapses are currently lacking, and development of such strategies are expected to aid clinical translation.

Supplemental Note 2:

10 Things to Know Before You Fall Victim to a Retinal Stem Cell Scam

1. **The Hope is real:** Stem cell technology has created exciting new possibilities for understanding and treating diseases that have perpetually plagued humankind. But these remain early days in the technology and we have an overarching obligation to be honest and transparent and to “first do no harm."

2. **The difference between Hope and Hype is a single letter and a compelling website.** Private stem cell clinics touting miracle cures can cause you to lose whatever vision you have left – or your entire eye – due to infection, tumor, or another catastrophic event. And even if the treatment causes no physical harm, it can result in significant financial damage, with costs often reaching into the tens of thousands of U.S. dollars.

3. **Confused? It’s NOT the fault of you or your family.** Stem cell technology is complicated and still relatively new, and there are a growing number of private clinics that are attempting to financially capitalize on patients’ desperation and confusion. You should know that in many cases, the “stem cells” that are now being transplanted in these for-profit clinics are from fat, bone marrow, peripheral blood, or another source that has no proven ability to replace missing retinal cells.

4. **Be highly skeptical of any stem cell therapy that requires you to pay a fee or that claims to be a cure-all.** Almost all valid stem cell therapies are still in the clinical trial stage, or even earlier. Ethical scientists will enroll patients in these trials without asking for, or accepting, payment (often, they pay YOU). If you have doubts, ask questions – and not just of the people trying to sell you the stem cell procedure or the folks they refer you to, since they have an inherent conflict of interest.

5. **In order to avoid scams, it is important to understand what the retina is….** The retina is actually a complex “layer cake,” with each layer containing specific types of cells that perform a precise job and connect to other cells to form a neural circuit. Deepest within the retina lies a layer of photoreceptors – rods and cones – that detect light and initiate a cascade of events that ultimately lead to our perception of vision, which occurs in the brain. Retinal pigmented epithelial cells, or RPE cells, do not detect light but rather help photoreceptors do their job. If you lose RPE cells, the photoreceptors they serve will eventually lose their ability to function and die as well.

6. **…and also to understand what happens when retinal cells die.** Some of the most devastating and incurable causes of blindness are rooted in the death of retinal cells, including photoreceptors and RPE cells (the layer which nourishes photoreceptors). These diseases include age-related macular degeneration (AMD), retinitis pigmentosa (RP), Stargardt disease, Best disease, Usher syndrome, and others. For the vast majority of those affected, there are no cures or successful treatments available.

7. **We are born with all the retinal “parts” we are ever going to have.** The human retina has no innate ability to replace these cells once they are lost. This is one reason why stem cells have drawn so much attention, since some types of stem cells (namely *pluripotent* stem cells, or PSCs) have the potential to provide replacement parts for the retina.

8. **Stem cell therapies may provide an option to replace the lost cells** by introducing new cells obtained from an outside source (retinal cell **replacement** through transplantation). Pluripotent stem cells (PSCs) – which are grown in the laboratory – can theoretically make any cell in the entire body. Many highly differentiated and specialized cell types, including photoreceptors and RPE cells, can now be produced from human PSCs in a reliable manner. There is another approach to replacing lost retinal cells, and that is to trick the retina into fixing itself (retinal **regeneration**), but that is a separate topic.

9. **The "installation challenge" is formidable for all cell types**, and scientists are working hard to develop effective methods of installing the new cells and getting them to connect properly and function within the retina itself. Of note, for-cost stem cell "clinics" will often just randomly inject their product into the middle of the eye, far away from the area where they would need to go if they truly could replace lost photoreceptors or RPE cells. Also, any such procedure has to be done by a skilled clinician, not a nurse or assistant.

10. **There is no magic to stem cells,** but there is a great deal of excellent, well-designed, and well-intentioned research being performed in the stem cell field. Stem cells have unique but variable properties that, *if thoughtfully tested and applied*, may be of considerable help to some patients in the foreseeable future. We're optimistic about this future…and you should be, too.

For a more detailed version of this article, please contact the McPherson Eye Research Institute at (608) 265-0690, vision.wisc.edu.

Supplemental References

1. Turner JE, Blair JR. Newborn rat retinal cells transplanted into a retinal lesion site in adult host eyes. *Brain Res*. 1986;391(1):91-104. doi:10.1016/0165-3806(86)90011-8

2. Ribeiro J, Procyk CA, West EL, et al. Restoration of visual function in advanced disease after transplantation of purified human pluripotent stem cell-derived cone photoreceptors. *Cell Rep*. 2021;35(3). doi:10.1016/j.celrep.2021.109022

3. Gasparini SJ, Llonch S, Borsch O, Ader M. Transplantation of photoreceptors into the degenerative retina: Current state and future perspectives. *Prog Retin Eye Res*. Published online November 13, 2018. doi:10.1016/j.preteyeres.2018.11.001

4. Nickerson PEB, Ortin-Martinez A, Wallace VA. Material Exchange in Photoreceptor Transplantation: Updating Our Understanding of Donor/Host Communication and the Future of Cell Engraftment Science. *Front Neural Circuits*. 2018;12:17. doi:10.3389/fncir.2018.00017

5. Gagliardi G, Ben M'Barek K, Chaffiol A, et al. Characterization and Transplantation of CD73-Positive Photoreceptors Isolated from Human iPSC-Derived Retinal Organoids. *Stem Cell Rep*. 2018;11(3):665-680. doi:10.1016/j.stemcr.2018.07.005

6. Aboualizadeh E, Phillips MJ, McGregor JE, et al. Imaging Transplanted Photoreceptors in Living Nonhuman Primates with Single-Cell Resolution. *Stem Cell Rep*. 2020;15(2):482-497. doi:10.1016/j.stemcr.2020.06.019

7. Laver CRJ, Matsubara JA. Structural divergence of essential triad ribbon synapse proteins among placental mammals – Implications for preclinical trials in photoreceptor transplantation therapy. *Exp Eye Res*. 2017;159:156-167. doi:10.1016/j.exer.2017.03.005

8. Carvalho LS, Pessoa DMA, Mountford JK, Davies WIL, Hunt DM. The Genetic and Evolutionary Drives behind Primate Color Vision. *Front Ecol Evol*. 2017;5. doi:10.3389/fevo.2017.00034
